# Quantum Geometric Moment Encodes Stacking Order of Moiré Matter

**DOI:** 10.1002/adma.202417682

**Published:** 2025-02-25

**Authors:** Surat Layek, Subhajit Sinha, Atasi Chakraborty, Ayshi Mukherjee, Heena Agarwal, Kenji Watanabe, Takashi Taniguchi, Amit Agarwal, Mandar M. Deshmukh

**Affiliations:** ^1^ Department of Condensed Matter Physics and Materials Science Tata Institute of Fundamental Research Homi Bhabha Road Mumbai 400005 India; ^2^ Institut für Physik Johannes Gutenberg Universität Mainz D‐55099 Mainz Germany; ^3^ Research Center for Functional Materials National Institute for Materials Science 1‐1 Namiki Tsukuba 305‐0044 Japan; ^4^ International Center for Materials Nanoarchitectonics National Institute for Materials Science 1‐1 Namiki Tsukuba 305‐0044 Japan; ^5^ Department of Physics Indian Institute of Technology Kanpur 208016 India

**Keywords:** Berry curvature dipole, 2D materials, graphene, moiré superlattices, nonlinear Hall transport

## Abstract

Exploring the topological characteristics of electronic bands is essential in condensed matter physics. Moiré materials featuring flat bands provide a versatile platform for engineering band topology and correlation effects. In moiré materials that break either time‐reversal symmetry or inversion symmetry or both, electronic bands exhibit Berry curvature hotspots. Different stacking orders in these materials result in varied Berry curvature distributions within the flat bands, even when the band dispersion remains similar. However, experimental studies probing the impact of stacking order on the quantum geometric quantities are lacking. 1.4° twisted double bilayer graphene (TDBG) facilitates two distinct stacking orders (AB‐AB, AB‐BA) and forms an inversion broken moiré superlattice with electrically tunable flat bands. The valley Chern numbers of the flat bands depend on the stacking order, and the nonlinear Hall (NLH) effect distinguishes the differences in Berry curvature dipole (BCD), the first moment of Berry curvature. The BCD exhibits antisymmetric behavior, flipping its sign with the polarity of the perpendicular electric field in AB‐AB TDBG, while it displays a symmetric behavior, maintaining the same sign regardless of the electric field's polarity in AB‐BA TDBG. This approach electronically detects stacking‐induced quantum geometry, while opening a pathway to quantum geometry engineering and detection.

## Graphical abstract

The Berry curvature dipole (BCD) changes across valley Chern transitions. A new electrical technique is developed to use such BCD sign flips for distinguishing the stacking order of a moiré material, namely twisted double bilayer graphene. Such a method, analogous to spectroscopic techniques, establishes a link between quantum geometry and stacking order, and is applicable to other moiré materials.

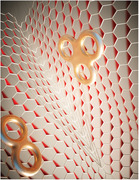

## 1 Introduction

Twistronics has emerged as a burgeoning field to engineer symmetry‐broken flat bands that can be tuned electrically and via other knobs.^[^ ^1^ ^]^ For example, magic‐angle twisted bilayer graphene hosts a plethora of tunable correlated phases such as superconductivity^[^ ^2^, ^3^ ^]^ and orbital ferromagnetism.^[^ ^4^, ^5^ ^]^ Recent advances in the field have drawn specific connections between electronic correlations in flat‐band systems and the underlying band topology. For instance, the superconductivity and superfluidity in the flat bands of twisted multilayer graphene systems are known to arise from the quantum geometry of the flat bands.^[^ ^6^ ^]^ It is also believed that fragile phases such as the fractional quantum anomalous Hall states^[^ ^7^, ^8^ ^]^ are better stabilized in bands with uniform Berry curvature^[^ ^9^ ^]^ and high Chern numbers.^[^ ^10^ ^]^ As a result, the topology of the flat bands can provide important information not only on the Berry curvature distribution but also on the accompanying correlated phases it is susceptible to host.

In this regard, twisted multilayer systems provide us with an additional knob to stack the multilayers with different stacking orders having distinct band topology. In some heterostructures, the stacking order leaves an imprint on the Berry curvature structure of the flat bands while keeping the energy dispersion of the bands similar. Engineering and studying such systems can help us determine the effects of the distinct topology of the bands on electronic transport. In addition, a change in stacking order across domain boundaries can induce unique topological electronic modes.^[^ ^11^ ^]^ Recently, domain boundaries across AB and BA domains in marginally twisted bilayer graphene have also been shown to host superconducting channels in the quantum Hall regime, highlighting the importance of studying the topology of distinct stacking orders of a system.^[^ ^12^ ^]^

In this work, we explore the stacking order‐induced differences in band topology by measuring the nonlinear Hall transport in twisted double bilayer graphene (TDBG). Owing to the moiré periodicity in TDBG, the *K* and the *K*′ moiré bands decouple. This decoupling allows a valley Chern number $C_{K}$ ($C_{K^{\prime }}$) to be defined for each moiré band of *K* (*K*′) valley.^[^ ^13^ ^]^ A nonzero $C_{K}$ ($C_{K^{\prime }}$) quantifies the nontrivial topology of the *K* (*K*′) moiré bands. In particular, the topological flat bands in TDBG^[^ ^14^, ^15^, ^16^, ^17^, ^18^, ^19^ ^]^ have non‐zero valley Chern numbers that depend on the stacking order–AB‐AB or AB‐BA. Tuning the valley Chern number, for example, via a perpendicular electric field,^[^ ^20^, ^21^, ^22^ ^]^ corresponds to changing the Z_2_ [$=(C_{K}-C_{K^{\prime }})/2$] topology of the system. Recent experiments^[^ ^23^ ^]^ and theoretical calculations^[^ ^24^, ^25^, ^26^ ^]^ have demonstrated that the Berry curvature dipole (BCD) senses topological transitions of the valley Chern type. Specifically, the BCD sign changes rapidly across specific topological Z_2_ transitions.^[^ ^23^, ^24^ ^]^ Here, using nonlinear Hall measurements at zero magnetic field, we study the effect of stacking order on the BCD of flat bands. We demonstrate that experimentally probing the BCD variation across valley Chern transitions can distinguish the stacking order induced distinct band topology in differently stacked heterostructures. We vary the polarity of the perpendicular electric field and find that the Berry curvature, and hence the BCD evolves differently depending on the stacking order of ≈1.4° TDBG. Our experiments show that nonlinear Hall transport can be utilized to detect the distinct stacking‐order induced BCD.

## 2 Results and Discussion

### 2.1 Band Structure Calculations of Twisted Double Bilayer Graphene

**Figure 1:**
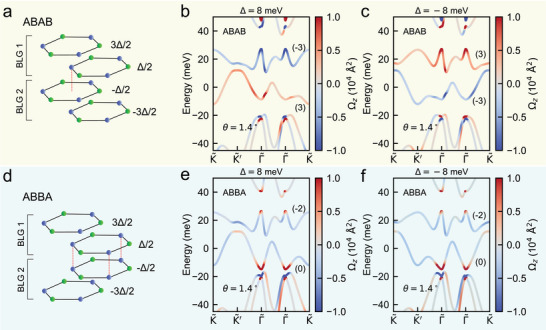
Change in Berry curvature distribution with perpendicular electric field in two distinct stacking orders of twisted double bilayer graphene (TDBG). a,d) Atomic arrangement in AB‐AB (a) and AB‐BA (d) stacked double bilayer graphene, before introducing any twist between the layers. The blue and green colors indicate the two different sublattices A and B. The dashed red line in (a) indicates that a sublattice of the top layer in BLG‐2 lies at the hexagon center of the bottom layer in BLG‐1 in the AB‐AB arrangement. In (d), the sublattices of the top layer in BLG‐2 and the bottom layer in BLG‐1 are aligned on top of each other. b,c) Band structure of 1.40° AB‐AB stacked TDBG for Δ = 8 meV (b) and Δ = −8 meV (c). The twist angle θ in TDBG is introduced between the two bilayers, BLG‐1 and BLG‐2. e,f) Band structure of 1.40° AB‐BA stacked TDBG for Δ = 8 meV (e) and Δ = −8 meV (f). The color indicates the Berry curvature (Ω_*z*_) of the bands. The valley Chern numbers are labeled for the flat bands. The Ω_*z*_ of the flat bands flip sign across (b) and (c), while it remains of the same sign across (e) and (f).

In TDBG, a Bernal (AB) bilayer graphene is stacked on another with a relative twist angle between them. Depending on how the second bilayer graphene is stacked (at an interlayer angle of θ or 180° + θ), TDBGs have two predominant stacking orders: AB‐AB‐stacked (**Figure** **1**a) TDBG,^[^ ^27^ ^]^ or AB‐BA‐stacked (Figure 1d) TDBG (see Section III, Supporting Information, for details on device fabrication). Our approach to distinguish how distinct stacking orders influence the band topology of TDBG is to pre‐determine the band structure for a particular twist angle and identify characteristic differences in Berry curvature and BCD. We calculate the band structure of 1.4° AB‐AB (Figure 1b,c) and AB‐BA (Figure 1e,f) TDBG for positive (Δ = 8 meV) and negative (Δ = −8 meV) interlayer potentials (Δ). We note three observations. i) For a fixed Δ, the valley Chern numbers of the flat bands are different for the two stacking orders, although the band dispersion is similar.^[^ ^21^ ^]^ ii) As we flip the polarity of Δ, the sign of the Berry curvature distribution in the flat bands of the AB‐AB TDBG flips (Figure 1b,c), whereas it remains unchanged in AB‐BA TDBG (Figure 1e,f). At the phenomenological level, the Berry curvature sign flip in AB‐AB TDBG is similar to the band inversion in AB bilayer graphene with Δ varying across Δ = 0 (we discuss this aspect later in Figure 5). iii) In the presence of time‐reversal symmetry, the valley Chern numbers *C*_*K*_ and $C_{K^{\prime }}$ are equal and opposite, resulting in a total Chern number ($C=C_{K}+C_{K^{\prime }}=0$) of zero, precluding any Berry curvature‐driven linear anomalous Hall response. This prompts a natural question: Can we distinguish the band topology of these two stacking orders in transport experiments. To address this, in the following we present linear and nonlinear transport experiments (backed by theoretical calculations) that probe the BCD in AB‐AB‐ and AB‐BA‐stacked TDBG, as perpendicular electric field switches polarity.

### 2.2 Linear and Nonlinear Hall transport

**Figure 2:**
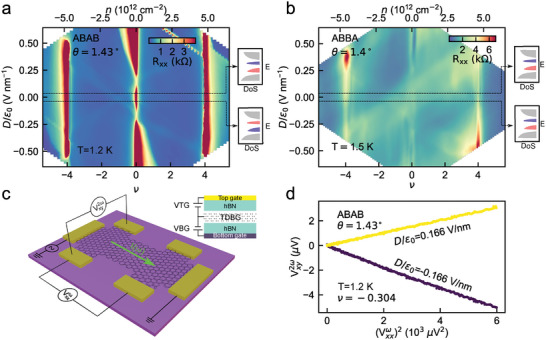
Transport characterization of the two stacking orders, AB‐AB and AB‐BA, in TDBG. a,b) Longitudinal resistance *R*_*xx*_ of 1.43° AB‐AB (a) and 1.40° AB‐BA (b) stacked TDBG devices as a function of filling factor (ν) and perpendicular electric field (*D*/ϵ_0_). The top axis in (a,b) indicates the charge density (*n*). The insets to the right in (a,b) are schematic representations to indicate the corresponding energy (E) versus density of states (DoS), close to the *K* valley, for two polarities of *D*/ϵ_0_. The colors indicate a nonzero Berry curvature of the flat bands that flip (does not flip) sign as the polarity of *D* is reversed in ≈1.4° AB‐AB (AB‐BA) TDBG. The measurement temperature *T* for (a) and (b) were 1.2 and 1.5 K, respectively. c) Measurement schematic for nonlinear Hall (NLH) voltage. An AC current, I(ω), is applied along the longitudinal direction of the device. The nonlinear Hall voltage, $V_{xy}^{2\omega }$ at twice the driving frequency (2ω) and the longitudinal voltage, $V_{xx}^{\omega }$, at the driving frequency (ω) are measured simultaneously while tuning the gate voltages to control the carrier density (*n*) and perpendicular electric field (*D*/ϵ_0_). The inset shows the cross‐sectional structure of the dual‐gated device, consisting of the TDBG layer encapsulated between hexagonal boron nitride (hBN) layers, with independent top and bottom gate electrodes. d) Variation of $V_{xy}^{2\omega }$ with ${\left(V_{xx}^{\omega }\right)}^{2}$ for a fixed filling factor ν = −0.304, for two different polarities of the perpendicular electric field *D*/ϵ_0_ in AB‐AB TDBG. A linear behavior of $V_{xy}^{2\omega }$ with ${\left(V_{xx}^{\omega }\right)}^{2}$ verifies the quadratic dependence of $V_{xy}^{2\omega }$ on current *I*(ω).

**Figure 3:**
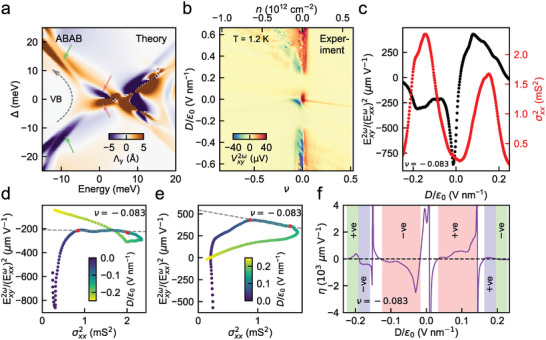
Sign flip in BCD with change in *D*/ϵ_0_ polarity for 1.43° AB‐AB TDBG. a) Calculated y‐component of BCD (Λ_*y*_) as a function of energy and interlayer potential (Δ) for 1.4° AB‐AB TDBG. The calculation was performed with an uniaxial strain of 0.2%, applied along the zig‐zag axis of a BLG in TDBG. The dashed arrow is a guide to the eye that traces the movement of the valence band in energy with Δ. The solid arrows show BCD sign changes when the polarity of Δ is reversed. b) Nonlinear Hall voltage ($V_{xy}^{2\omega }$) as a function of filling factor (ν) (corresponding *n* is shown on the top‐axis) and perpendicular electric field (*D*/ϵ_0_) for 1.43° AB‐AB twisted TDBG. c) $\frac{E_{xy}^{2\omega }}{{\left(E_{xx}^{\omega }\right)}^{2}}$ (left axis; black data points) and $\sigma_{xx}^{2}$ (right axis; red data points) as a function of *D*/ϵ_0_ for a filling ν = −0.083 in the valence band. d,e) $\frac{E_{xy}^{2\omega }}{{\left(E_{xx}^{\omega }\right)}^{2}}$ as a function of $\sigma_{xx}^{2}$ at ν = −0.083, where *D*/ϵ_0_ is varied parametrically for *D* < 0 (d) and *D* > 0 (e). The red dots indicate the *D*/ϵ_0_ range within which the linear fit of the form $\frac{E_{xy}^{2\omega }}{{\left(E_{xx}^{\omega }\right)}^{2}}$ = ζ$\sigma_{xx}^{2}$+η is performed (−0.066 to −0.116 V nm^−1^ in (d), and 0.078 to 0.124 V nm^−1^ in (e)). The intercept η changes sign across (d) and (e) as *D* changes sign. f) The extracted local intercept η as a function of *D*/ϵ_0_ for ν = −0.083. The colors indicate the different *D*/ϵ_0_ ranges where η flips sign across *D* = 0. This captures the sign change of BCD on reversing the polarity of *D* in the AB‐AB TDBG. The measurements were performed at *T* = 1.2 K.

**Figure 4:**
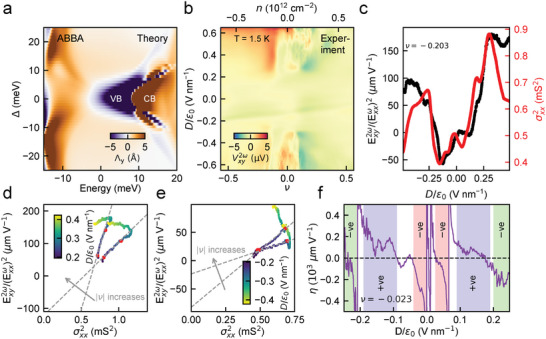
Sign of BCD is intact with change in *D*/ϵ_0_ polarity for 1.40° AB‐BA TDBG. a) Calculated y‐component of BCD (Λ_*y*_) as a function of energy and interlayer potential (Δ) for 1.40° AB‐BA TDBG. The calculation was performed with the same strain parameters as in Figure 3a. b) Nonlinear Hall voltage ($V_{xy}^{2\omega }$) as a function of filling factor (ν) (corresponding *n* is shown on the top‐axis) and perpendicular electric field (*D*/ϵ_0_) for 1.40° AB‐BA twisted TDBG at *T* = 1.5 K. c) $\frac{E_{xy}^{2\omega }}{{\left(E_{xx}^{\omega }\right)}^{2}}$ (left axis; black data points) and $\sigma_{xx}^{2}$ (right axis; red data points) as a function of *D*/ϵ_0_ for a filling ν = −0.203 in the valence band. d,e) $\frac{E_{xy}^{2\omega }}{{\left(E_{xx}^{\omega }\right)}^{2}}$ as a function of $\sigma_{xx}^{2}$ where *D*/ϵ_0_ is varied parametrically for *D* > 0 (d) and *D* < 0 (e) for the fixed filling factors ν = −0.203, −0.248 in the valence band. The dashed gray line indicates a linear fit of the form $\frac{E_{xy}^{2\omega }}{{\left(E_{xx}^{\omega }\right)}^{2}}$ = ζ$\sigma_{xx}^{2}$+η for the two fillings and the red dots indicate the fitting range. The intercept η does not change sign across (d) and (e) even though *D* changes sign. f) The extracted local intercept η as a function of *D*/ϵ_0_ for ν = −0.023. The colors indicate the different *D*/ϵ_0_ ranges where η does not flip sign across *D* = 0. This captures the fact that the BCD does not change sign in the AB‐BA TDBG on reversing the polarity of *D*. The measurements were performed at *T* = 1.5 K.

In **Figure** **2**a,b, we show the measured longitudinal resistance *R*_*xx*_ as a function of the filling factor ν = 4*n*/*n*_S_ and perpendicular electric field *D*/ϵ_0_ (the dual‐gated geometry in our devices allow independent control of the charge density *n* and *D*/ϵ_0_, see Section IV.1, Supporting Information, for details) for AB‐AB TDBG and AB‐BA TDBG, respectively. Here, *n*_*S*_ = 4.80 × 10^12^ cm^−2^ (*n*_*S*_ = 4.62 × 10^12^ cm^−2^) is the charge density required to fill or empty a flat band completely in AB‐AB (AB‐BA) TDBG. The twist angles of the two distinct stacking orders (see Section IV.1, Supporting Information, for the twist angle estimations), are 1.43° (AB‐AB) and 1.40° (AB‐BA) (see Section VIII.2, Supporting Information, for AB‐AB TDBG device‐2 with a twist angle of 1.1°). The high values of *R*_*xx*_ at *n* = ±*n*_*S*_ indicate the presence of moiré gaps. For fillings close to ν = 0, the *R*_*xx*_ shows a minimum as |*D*|/ϵ_0_ is increased in both AB‐AB and AB‐BA TDBG, corresponding to a peak in conductivity squared $\sigma_{xx}^{2}$ in **Figure** **3**c and **Figure** **4**c, respectively. Such a feature is attributed to a gap closing and reopening transition^[^ ^18^ ^]^ at a nonzero *D*/ϵ_0_ (see Section V, Supporting Information, for the temperature dependence of *R*_*xx*_ at the charge neutrality gap) and is also reproduced in our theoretical calculations (see Figure S3, Supporting Information, for a band touching and reopening transition). The subtle differences in strain and twist angle of the two devices can possibly cause a difference in the measured value of the conductivity across the two devices.

Recently, there has been a growing interest in studying the nonlinear effects in materials, owing to their connection with the quantum geometry of bands.^[^ ^28^, ^29^, ^30^ ^]^ In the presence of time‐reversal symmetry, broken inversion symmetry is essential for nonzero Berry curvature. In multilayer systems such as bilayer graphene, the perpendicular electric field breaks the inversion symmetry and introduces a nonzero Berry curvature at the band edge. A broken C_3_ symmetry (such as due to non‐zero in‐plane strain in moiré superlattices,^[^ ^31^, ^32^, ^33^ ^]^ see Section IV.2, Supporting Information, for evidence of strain in our TDBG device) together with broken inversion symmetry, creates a non‐uniform Berry curvature distribution in *k*‐space resulting in a nonzero BCD, $\Lambda_{\alpha }=\sum_{n}\int_{\mathrm{mBZ}}\frac{dk}{{\left(2\pi \right)}^{2}}\Omega_{z}^{n}\frac{\partial \varepsilon_{k}^{n}}{\hbar \partial k_{\alpha }}\frac{\partial f(\varepsilon_{k}^{n})}{\partial \varepsilon_{k}^{n}}$. Here, the integral is carried over the moiré Brillouin zone (mBZ), α stands for the spatial index (*x*, *y*), $\varepsilon_{k}^{n}$ is the energy of the *n* ^*th*^ band, $f(\varepsilon_{k}^{n})$ is the Fermi–Dirac function, and a sum over all the bands crossing the Fermi energy is implied. A nonzero BCD generates a second‐order nonlinear Hall response ${\vec{j}}^{2\omega }\propto \hat{z}\times {\vec{E}}^{\omega }\left(\vec{\Lambda }\cdot {\vec{E}}^{\omega }\right)$ that is detected by measuring^[^ ^34^ ^]^ the nonlinear Hall (NLH) voltage $V_{xy}^{2\omega }$. Figure 2c shows our schematic to measure the NLH voltage. The linear dependence of $V_{xy}^{2\omega }$ on ${\left(V_{xx}^{\omega }\right)}^{2}$ in Figure 2d confirms the characteristic second‐order nature of the measured $V_{xy}^{2\omega }$ in the AB‐AB TDBG device (see Section VI, Supporting Information, for additional characterization of nonlinear voltage in the TDBG devices). NLH response has been investigated in transition metal dichalcogenides (TMDCs),^[^ ^35^, ^36^, ^37^, ^38^, ^39^, ^40^, ^41^, ^42^ ^]^ corrugated graphene,^[^ ^43^ ^]^ 3D systems,^[^ ^24^, ^44^ ^]^ and recently in few moiré superlattices owing to both BCD^[^ ^23^, ^45^, ^46^ ^]^ and scattering^[^ ^47^, ^48^ ^]^ mechanisms. Hence it is important to devise a pathway forward to systematically analyze and segregate the intrinsic and extrinsic mechanisms. Next, we systematically compare the measured $V_{xy}^{2\omega }$ versus *D*/ϵ_0_ dependence across a change in the polarity of *D*/ϵ_0_, which distinguishes the band topology of AB‐AB and AB‐BA TDBG.

### 2.3 Berry Curvature Dipole Calculations and Scaling Analysis

In Figure 3a, we show the calculated Λ_*y*_ for 1.4° AB‐AB TDBG as a function of energy and Δ (where Δ is proportional to *D*/ϵ_0_; see Figure S4 (Supporting Information) for the BCD dependence on a greater energy range). The choice of a twist angle of 1.4° allows us to explore the BCD of isolated flat bands. We see that as Δ is flipped, Λ_*y*_ changes its sign. The sign reversal is most apparent for the valence band. In Section I (Figures S1 and S2, Supporting Information), we show the band structure calculations with a nonzero strain and plot the corresponding BCD versus energy lineslices for different Δ. As Δ is varied and flipped, the flat bands undergo band touchings and consequently, the Berry curvature distribution and valley Chern numbers change reflecting in the sign change of BCD.

To experimentally detect this sign reversal of BCD, we measure $V_{xx}^{\omega }$ (Figure 2a shows the corresponding *R*_*xx*_ = $V_{xx}^{\omega }$/*I*, where *I* is the channel current) and $V_{xy}^{2\omega }$ (Figure 3b) as a function of the perpendicular electric field *D*/ϵ_0_ and fillings close to the charge neutrality point ν = 0. $V_{xx}^{\omega }$ and $V_{xy}^{2\omega }$ correspond to the linear $E_{xx}^{\omega }$(=$V_{xx}^{\omega }$/*L*) and nonlinear $E_{xy}^{2\omega }$(=$V_{xy}^{2\omega }$/*w*) in‐plane electric fields, where *L* and *w* are the length and width of the device, respectively. In general, the measured $V_{xy}^{2\omega }$ contains the intrinsic BCD contribution along with extrinsic contributions such as the skew scattering and side‐jump mechanisms. A way forward to segregate the intrinsic BCD contribution from other extrinsic contributions is to study the linear scaling of the form^[^ ^23^, ^35^, ^49^ ^]^ $\frac{E_{xy}^{2\omega }}{{\left(E_{xx}^{\omega }\right)}^{2}}$ = ζ$\sigma_{xx}^{2}$+η (over a small window of *D*/ϵ_0_), where ζ and η are the slope and intercept, respectively (see Section VII.1, Supporting Information, for details). Here, the intercept η is used as an order of magnitude estimation^[^ ^35^ ^]^ of BCD∼η*E*_*F*_/*e*, where *E*_*F*_ is the Fermi energy. Figure 3c shows a representative lineslice of the $\frac{E_{xy}^{2\omega }}{{\left(E_{xx}^{\omega }\right)}^{2}}$ and $\sigma_{xx}^{2}$ with *D*/ϵ_0_, for a fixed filling of ν = −0.083 in the valence band. Figure 3d,e probes the scaling relation for ‐ve and +ve values of *D*, respectively. Here, *D* is varied as a parameter to probe the linear scaling relation (see Section VII.2, Supporting Information). We first probe the scaling for both polarities of the perpendicular displacement field *D* for |*D*| < |D*|, where σ_*xx*_ is maximum at |D*|/ϵ_0_ ≈ 0.16 V nm^−1^ corresponding to the gap closing discussed earlier. We find that the intercept η changes sign when fitted linearly within a similar |*D*|/ϵ_0_ range across Figure 3d,e. This choice of |*D*|/ϵ_0_ range guarantees that the analysis is performed in a |*D*|/ϵ_0_ range within which no drastic band structure changes such as a gap closing and reopening occurs. Although our devices at this twist angle show non‐zero $V_{xx}^{2\omega }$ (see Section VI, Supporting Information) that is typically attributed to extrinsic scattering mechanisms,^[^ ^48^ ^]^ a sign change in intercept (η) with *D*/ϵ_0_ cannot be explained via scattering mechanisms alone.^[^ ^36^ ^]^ The sign change in intercept (η) agrees with our calculated BCD sign reversal with the polarity of Δ in the valence band (Figure 3a), and captures the intrinsic contribution at this filling (see Section VIII.1, Supporting Information, for similar results at other fillings).

We now focus on how reversing the polarity of *D*/ϵ_0_ affects the BCD sign, for an extended *D*/ϵ_0_ range. We probe the local intercept η as a function of *D*/ϵ_0_ (here, η is defined locally for a small moving window of *D*/ϵ_0_; see Section VII.3, Supporting Information, for details of the analysis) across band‐touching transitions in Figure 3f. The intercept η changes sign for the similar magnitude range of *D*/ϵ_0_ but opposite polarity, indicated by the same color. The opposite signs of intercepts for the opposite polarity of *D*/ϵ_0_ indicate that the BCD flips sign once the electric field polarity is reversed in AB‐AB TDBG (Figure S4b,c, Supporting Information, shows that the dependence of the valley Chern number with Δ is anti‐symmetric). Interestingly, we also see a sharp change in the intercept η in the white‐colored regions across valley Chern transitions. The BCD is theoretically known to increase and switch rapidly across a band touching topological transition,^[^ ^24^ ^]^ which further confirms the intrinsic‐dominated origin of the measured η. We next carry out the same analysis for a 1.4° AB‐BA TDBG device to examine the BCD evolution when the polarity of the perpendicular electric field flips.

**Figure 5:**
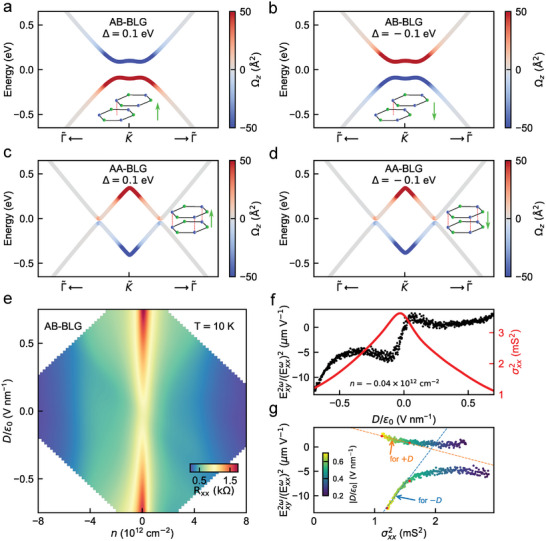
BCD sign reversal in AB‐stacked bilayer graphene. a,b) Band structure of AB‐bilayer graphene for Δ = 0.1 eV (a) and Δ = −0.1 eV (b). Insets show the atomic arrangement of Bernal (AB) bilayer graphene, where the green arrow indicates the direction of applied interlayer potential Δ (a nonzero Δ translates to an applied perpendicular *D*/ϵ_0_ in experiments). The Berry curvature of each band (indicated by color) changes sign as the sign of Δ (polarity of *D*) is flipped. c,d) Band structure of AA‐bilayer graphene for Δ = 0.1 eV (c) and Δ = −0.1 eV (d), with an induced gap (see Section II.1, Supporting Information) to have nonzero Berry curvature. Insets show the atomic arrangement of AA‐stacked bilayer graphene, where the green arrow indicates the direction of applied Δ. The Berry curvature of each band (indicated by color) does not change sign as the sign of Δ is flipped. e) Longitudinal resistance *R*_*xx*_ as a function of *n* and *D*/ϵ_0_ in AB‐stacked bilayer graphene. f) $\frac{E_{xy}^{2\omega }}{{\left(E_{xx}^{\omega }\right)}^{2}}$ (left axis; black data points) and $\sigma_{xx}^{2}$ (right axis; red data points) as a function of *D*/ϵ_0_ for a fixed charge density *n* = −0.04 × 10^12^ cm^−2^ close to the charge neutrality gap. g) $\frac{E_{xy}^{2\omega }}{{\left(E_{xx}^{\omega }\right)}^{2}}$ as a function of $\sigma_{xx}^{2}$ where *D*/ϵ_0_ is varied parametrically, for *D* > 0 (upper plot) and *D* < 0 (lower plot) for the fixed charge density *n* = −0.04 × 10^12^ cm^−2^. The dashed orange (blue) line indicates a linear fit of the form $\frac{E_{xy}^{2\omega }}{{\left(E_{xx}^{\omega }\right)}^{2}}$ = ζ$\sigma_{xx}^{2}$+η, performed for the high positive (negative) *D*/ϵ_0_ range, and the red dots indicate the fitting range. The intercept η is positive (negative) for *D* > 0 (*D* < 0), thus indicating that the BCD of AB‐bilayer graphene changes sign as the perpendicular electric field is flipped. The measurements in (e–g) were performed at 10 K.

In Figure 4a, we show the theoretically calculated BCD, Λ_*y*_, in the flat bands of 1.4° AB‐BA TDBG. In contrast to 1.4° AB‐AB TDBG discussed in Figure 3a, we do not see a sign change in the calculated BCD as the polarity of Δ is reversed. This is analogous to an AA‐bilayer graphene system (discussed later in **Figure** **5**c,d). To experimentally validate this observation, we measured the $V_{xy}^{2\omega }$ in AB‐BA TDBG with a twist angle of 1.40° at 1.5 K in Figure 4b. Figure 4c shows a representative lineslice of the $\frac{E_{xy}^{2\omega }}{{\left(E_{xx}^{\omega }\right)}^{2}}$ and $\sigma_{xx}^{2}$ with *D*/ϵ_0_, for a fixed filling of ν = −0.203 in the valence band to test the scaling relation. Figure 4d,e probe the scaling relation for +ve and ‐ve values of *D*, respectively, where *D* is varied as a parameter for two fixed fillings in the valence band (see Section IX, Supporting Information, for other fillings ν). Interestingly, in this case, the intercept η does not change sign across a change in *D* polarity (Figure 4d,e), in agreement with the BCD calculation presented in Figure 4a. The decrease in intercept η with increasing |ν| placed inside the flat valance band further agrees with AB‐BA TDBG studied in Zhong et al.,^[^ ^50^ ^]^ and indicates the domination of intrinsic contribution in this ν range. The fact that the BCD in the AB‐BA TDBG does not flip with a change in polarity of *D* is also evident in the dependence of the local intercept η over an extended *D*/ϵ_0_ range in Figure 4f (Figure S4e,f, Supporting Information, shows that the dependence of the valley Chern number with Δ is symmetric for 1.4° AB‐BA TDBG).

### 2.4 Nonlinear Hall Transport in Bilayer Graphene

A simpler system in which the BCD sign reversal with the reversal of *D* polarity is expected, analogous to AB‐AB TDBG discussed earlier in Figure 3, is the Bernal (AB‐stacked) bilayer graphene (BLG). Figure 5a,b, shows the low‐energy bandstructure of AB‐BLG, where the Berry curvature, and consequently the BCD, of the bands flip with the polarity of Δ (see Sections II.2 and II.3, Supporting Information, for details of theoretical calculations). To experimentally verify this BCD sign reversal, we fabricated a dual‐gated bilayer graphene device. Figure 5e shows the measured *R*_*xx*_ with *n* and *D*/ϵ_0_. We note that at the charge neutrality point (*n* = 0), the *R*_*xx*_ increases as the magnitude of the perpendicular electric field |*D*|/ϵ_0_ is increased. This is due to the band gap opening with *D*/ϵ_0_ at charge neutrality in BLG, consistent with earlier works.^[^ ^51^ ^]^ In Figure 5f, we plot the measured $\frac{E_{xy}^{2\omega }}{{\left(E_{xx}^{\omega }\right)}^{2}}$ and $\sigma_{xx}^{2}$ as a function of *D*/ϵ_0_ close to the charge neutrality point. To extract the intrinsic BCD contribution at the band edge, in Figure 5g we plot $\frac{E_{xy}^{2\omega }}{{\left(E_{xx}^{\omega }\right)}^{2}}$ with $\sigma_{xx}^{2}$ parametrically as a function of *D*/ϵ_0_, for both the polarities of *D*/ϵ_0_. We fit the $\frac{E_{xy}^{2\omega }}{{\left(E_{xx}^{\omega }\right)}^{2}}$ versus $\sigma_{xx}^{2}$ dependence with the linear scaling relation used earlier, in the high *D*/ϵ_0_ regime where the variation of Berry curvature with *D*/ϵ_0_ is relatively low (see Section II.4, Supporting Information). We find that the intercept η of the linear scaling (dashed line in Figure 5g), and thus the BCD, indeed flips with a reversal in *D*/ϵ_0_ polarity, in analogy to the BCD sign reversal of AB‐AB TDBG discussed in Figure 3. On the contrary, the calculated Berry curvature of AA‐stacked BLG does not flip as the polarity of *D*/ϵ_0_ is flipped (Figure 5c,d), analogous to ABBA‐TDBG discussed in Figure 4. Together, the experimental observation along with the theoretical calculations on BLG demonstrate that the nonlinear Hall transport is sensitive to the BCD sign reversal with the *D*/ϵ_0_ polarity in AB‐BLG.

## 3 Conclusion

In summary, we find that TDBG has two distinct stacking orders, namely AB‐AB and AB‐BA, with similar band dispersion but different valley Chern numbers, most apparent when the flat bands are isolated from the remote bands. The parameter space for tuning bands in TDBG is substantially large; it comprises of twist angle θ, strain %, and *D*/ϵ_0_. In particular, *D*/ϵ_0_ tunes the valley Chern numbers of the flat bands in TDBG. We demonstrate a way to electrically distinguish the flat band quantum geometry of the two distinct stacking orders of ≈1.4° TDBG that have different valley Chern numbers, by studying the nonlinear Hall voltage as a function of the perpendicular electric field (*D*/ϵ_0_). Our central observation is that the sign of BCD is odd as a function of the perpendicular electric field for one stacking (AB‐AB) and even for the other stacking (AB‐BA). Our study offers an example of how the stacking of layers provides insight into the distinct topological structure of electronic bands, using the nonlinear Hall effect. Our work motivates the use of nonlinear Hall transport to probe and identify differently stacked twisted heterostructures, such as twisted transition metal dichalcogenides,^[^ ^52^ ^]^ or other 2D materials.

## Conflict of Interest

The authors declare no conflict of interest.

## Author Contributions

S.L., S.S., and A.C., contributed equally to this work. S.L. and S.S fabricated the devices. A.M. and H.A. helped in fabrication. S.S. and S.L. did the measurements and analyzed the data. A.C. and A.A. did the theoretical calculations. K.W. and T.T. grew the hBN crystals. S.S., S.L., A.C., and M.M.D. wrote the manuscript with inputs from all authors. M.M.D. supervised the project.

## Supporting information

Supporting Information

## Data Availability

The data that support the findings of this study are available from the corresponding author upon reasonable request.

## References

[1] P. C. Adak , S. Sinha , A. Agarwal , M. M. Deshmukh , Nat. Rev. Mater. 2024, 1.

[2] Y. Cao , V. Fatemi , S. Fang , K. Watanabe , T. Taniguchi , E. Kaxiras , P. Jarillo‐Herrero , Nature 2018, 556, 43. 29512651 10.1038/nature26160

[3] X. Lu , P. Stepanov , W. Yang , M. Xie , M. A. Aamir , I. Das , C. Urgell , K. Watanabe , T. Taniguchi , G. Zhang , A. Bachtold , A. H. MacDonald , D. K. Efetov , Nature 2019, 574, 653. 31666722 10.1038/s41586-019-1695-0

[4] A. L. Sharpe , E. J. Fox , A. W. Barnard , J. Finney , D. Goldhaber‐Gordon , Science 2019, 365, 605. 31346139 10.1126/science.aaw3780

[5] M. Serlin , C. L. Tschirhart , H. Polshyn , Y. Zhang , A. F. Young , Science 2020, 367, 900. 31857492 10.1126/science.aay5533

[6] Törmä, P., S. Peotta , B. A. Bernevig , Nat. Rev. Phys. 2022, 4, 528.

[7] J. Cai , E. Anderson , C. Wang , X. Zhang , X. Liu , W. Holtzmann , Y. Zhang , F. Fan , T. Taniguchi , K. Watanabe , Y. Ran , T. Cao , L. Fu , D. Xiao , W. Yao , X. Xu , Nature 2023, 622, 63. 37315640 10.1038/s41586-023-06289-w

[8] Z. Lu , T. Han , Y. Yao , A. P. Reddy , J. Yang , J. Seo , K. Watanabe , T. Taniguchi , L. Fu , L. Ju , Nature 2024, 626, 759. 38383622 10.1038/s41586-023-07010-7

[9] Y. Xie , A. T. Pierce , J. M. Park , D. E. Parker , E. Khalaf , P. Ledwith , Y. Cao , S. H. Lee , S. Chen , P. R. Forrester , K. Watanabe , T. Taniguchi , A. Vishwanath , P. Jarillo‐Herrero , A. Yacoby , Nature 2021, 600, 439. 34912084 10.1038/s41586-021-04002-3 PMC8674130

[10] J. Herzog‐Arbeitman , Y. Wang , J. Liu , P. M. Tam , Z. Qi , Y. Jia , D. K. Efetov , O. Vafek , N. Regnault , Phys. Rev. B 2024, 109, 205122.

[11] L. Ju , Z. Shi , N. Nair , Y. Lv , C. Jin , J. V. Jr, C. Ojeda‐Aristizabal , H. A. Bechtel , M. C. Martin , A. Zettl , J. Analytis , F. Wang , Nature 2015, 520, 650. 25901686 10.1038/nature14364

[12] J. Barrier , M. Kim , R. K. Kumar , N. Xin , P. Kumaravadivel , L. Hague , E. Nguyen , A. I. Berdyugin , C. Moulsdale , V. V. Enaldiev , J. R. Prance , F. H. L. Koppens , R. V. Gorbachev , K. Watanabe , T. Taniguchi , L. I. Glazman , I. V. Grigorieva , V. I. Fal'ko , A. K. Geim , Nature 2024, 628, 741. 38658686 10.1038/s41586-024-07271-w

[13] J. C. W. Song , P. Samutpraphoot , L. S. Levitov , Proc. Natl. Acad. Sci. USA 2015, 112, 10879. 26286992 10.1073/pnas.1424760112 PMC4568281

[14] G. W. Burg , J. Zhu , T. Taniguchi , K. Watanabe , A. H. MacDonald , E. Tutuc , Phys. Rev. Lett. 2019, 123, 197702. 31765206 10.1103/PhysRevLett.123.197702

[15] C. Shen , Y. Chu , Q. Wu , N. Li , S. Wang , Y. Zhao , J. Tang , J. Liu , J. Tian , K. Watanabe , T. Taniguchi , R. Yang , Z. Y. Meng , D. Shi , O. V. Yazyev , G. Zhang , Nat. Phys. 2020, 16, 520.

[16] S. Sinha , P. C. Adak , Surya Kanthi R. S., B. L. Chittari , L. D. V. Sangani , K. Watanabe , T. Taniguchi , J. Jung , M. M. Deshmukh , Nat. Commun. 2020, 11, 5548. 33144578 10.1038/s41467-020-19284-w PMC7641251

[17] Y. Cao , D. Rodan‐Legrain , O. Rubies‐Bigorda , J. M. Park , K. Watanabe , T. Taniguchi , P. Jarillo‐Herrero , Nature 2020, 583, 215. 32499644 10.1038/s41586-020-2260-6

[18] X. Liu , Z. Hao , E. Khalaf , J. Y. Lee , Y. Ronen , H. Yoo , D. H. Najafabadi , K. Watanabe , T. Taniguchi , A. Vishwanath , P. Kim , Nature 2020, 583, 221. 32641816 10.1038/s41586-020-2458-7

[19] Y. Wang , J. Herzog‐Arbeitman , G. W. Burg , J. Zhu , K. Watanabe , T. Taniguchi , A. H. MacDonald , B. A. Bernevig , E. Tutuc , Nat. Phys. 2022, 18, 48.

[20] Y.‐H. Zhang , D. Mao , Y. Cao , P. Jarillo‐Herrero , T. Senthil , Phys. Rev. B 2019, 99, 075127.

[21] M. Koshino , Phys. Rev. B 2019, 99, 235406.

[22] P. C. Adak , S. Sinha , D. Giri , D. K. Mukherjee , Chandan, L. D. V. Sangani , S. Layek , A. Mukherjee , K. Watanabe , T. Taniguchi , H. A. Fertig , A. Kundu , M. M. Deshmukh , Nat. Commun. 2022, 13, 7781. 36526625 10.1038/s41467-022-35421-z PMC9758152

[23] S. Sinha , P. C. Adak , A. Chakraborty , K. Das , K. Debnath , L. D. V. Sangani , K. Watanabe , T. Taniguchi , U. V. Waghmare , A. Agarwal , M. M. Deshmukh , Nat. Phys. 2022, 18, 765.

[24] J. I. Facio , D. Efremov , K. Koepernik , J.‐S. You , I. Sodemann , J. van den Brink , Phys. Rev. Lett. 2018, 121, 246403. 30608737 10.1103/PhysRevLett.121.246403

[25] J.‐X. Hu , C.‐P. Zhang , Y.‐M. Xie , K. T. Law , Commun. Phys. 2022, 5, 1.

[26] A. Chakraborty , K. Das , S. Sinha , P. C. Adak , M. M. Deshmukh , A. Agarwal , 2D Mater. 2022, 9, 045020.

[27] P. C. Adak , S. Sinha , U. Ghorai , L. D. V. Sangani , K. Watanabe , T. Taniguchi , R. Sensarma , M. M. Deshmukh , Phys. Rev. B 2020, 101, 125428.

[28] Q. Ma , A. G. Grushin , K. S. Burch , Nat. Mater. 2021, 20, 1601. 34127824 10.1038/s41563-021-00992-7

[29] Z. Z. Du , H.‐Z. Lu , X. C. Xie , Nat. Rev. Phys. 2021, 3, 744.

[30] Q. Fu , X. Cong , X. Xu , S. Zhu , X. Zhao , S. Liu , B. Yao , M. Xu , Y. Deng , C. Zhu , X. Wang , L. Kang , Q. Zeng , M.‐L. Lin , X. Wang , B. Tang , J. Yang , Z. Dong , F. Liu , Q. Xiong , J. Zhou , Q. Wang , X. Li , P.‐H. Tan , B. K. Tay , Z. Liu , Adv. Mater. 2023, 35, 2306330. 10.1002/adma.202306330 37737448

[31] N. P. Kazmierczak , M. V. Winkle , C. Ophus , K. C. Bustillo , S. Carr , H. G. Brown , J. Ciston , T. Taniguchi , K. Watanabe , D. K. Bediako , Nat. Mater. 2021, 20, 956. 33859383 10.1038/s41563-021-00973-w

[32] L. J. McGilly , A. Kerelsky , N. R. Finney , K. Shapovalov , E.‐M. Shih , A. Ghiotto , Y. Zeng , S. L. Moore , W. Wu , Y. Bai , K. Watanabe , T. Taniguchi , M. Stengel , L. Zhou , J. Hone , X. Zhu , D. N. Basov , C. Dean , C. E. Dreyer , A. N. Pasupathy , Nat. Nanotechnol. 2020, 15, 580. 32572229 10.1038/s41565-020-0708-3

[33] Y. Li , X. Wang , D. Tang , X. Wang , K. Watanabe , T. Taniguchi , D. R. Gamelin , D. H. Cobden , M. Yankowitz , X. Xu , J. Li , Adv. Mater. 2021, 33, 2105879. 10.1002/adma.202105879 34632646

[34] L. Fu , I. Sodemann , Phys. Rev. Lett. 2015, 115, 216806. 26636867 10.1103/PhysRevLett.115.216806

[35] K. Kang , T. Li , E. Sohn , J. Shan , K. F. Mak , Nat. Mater. 2019, 18, 324. 30804510 10.1038/s41563-019-0294-7

[36] Q. Ma , S.‐Y. Xu , H. Shen , D. MacNeill , V. Fatemi , T.‐R. Chang , A. M. Mier Valdivia , S. Wu , Z. Du , C.‐H. Hsu , S. Fang , Q. D. Gibson , K. Watanabe , T. Taniguchi , R. J. Cava , E. Kaxiras , H.‐Z. Lu , H. Lin , L. Fu , N. Gedik , P. Jarillo‐Herrero , Nature 2019, 565, 337. 30559379 10.1038/s41586-018-0807-6

[37] O. O. Shvetsov , V. D. Esin , A. V. Timonina , N. N. Kolesnikov , E. V. Deviatov , JETP Lett. 2019, 109, 715.

[38] J. Xiao , Y. Wang , H. Wang , C. D. Pemmaraju , S. Wang , P. Muscher , E. J. Sie , C. M. Nyby , T. P. Devereaux , X. Qian , X. Zhang , A. M. Lindenberg , Nat. Phys. 2020, 16, 1028.

[39] A. Tiwari , F. Chen , S. Zhong , E. Drueke , J. Koo , A. Kaczmarek , C. Xiao , J. Gao , X. Luo , Q. Niu , Y. Sun , B. Yan , L. Zhao , A. W. Tsen , Nat. Commun. 2021, 12, 2049. 33824340 10.1038/s41467-021-22343-5 PMC8024290

[40] D. Kumar , C.‐H. Hsu , R. Sharma , T.‐R. Chang , P. Yu , J. Wang , G. Eda , G. Liang , H. Yang , Nat. Nanotechnol. 2021, 16, 421. 33495620 10.1038/s41565-020-00839-3

[41] J. Son , K.‐H. Kim , Y. Ahn , H.‐W. Lee , J. Lee , Phys. Rev. Lett. 2019, 123, 036806. 31386425 10.1103/PhysRevLett.123.036806

[42] M. Huang , Z. Wu , J. Hu , X. Cai , E. Li , L. An , X. Feng , Z. Ye , N. Lin , K. T. Law , N. Wang , Natl. Sci. Rev. 2022, nwac232. 37180357 10.1093/nsr/nwac232 PMC10171643

[43] S.‐C. Ho , C.‐H. Chang , Y.‐C. Hsieh , S.‐T. Lo , B. Huang , T.‐H.‐Y. Vu , C. Ortix , T.‐M. Chen , Nat. Electron. 2021, 4, 116.

[44] Y. Zhang , Y. Sun , B. Yan , Phys. Rev. B 2018, 97, 041101.

[45] M. Huang , Z. Wu , X. Zhang , X. Feng , Z. Zhou , S. Wang , Y. Chen , C. Cheng , K. Sun , Z. Y. Meng , N. Wang , Phys. Rev. Lett. 2023, 131, 066301. 37625039 10.1103/PhysRevLett.131.066301

[46] S. Datta , S. Bhowmik , H. Varshney , K. Watanabe , T. Taniguchi , A. Agarwal , U. Chandni , Nano Lett. 2024, 24, 9520. 39058474 10.1021/acs.nanolett.4c01946

[47] J. Duan , Y. Jian , Y. Gao , H. Peng , J. Zhong , Q. Feng , J. Mao , Y. Yao , Phys. Rev. Lett. 2022, 129, 186801. 36374703 10.1103/PhysRevLett.129.186801

[48] P. He , G. K. W. Koon , H. Isobe , J. Y. Tan , J. Hu , A. H. C. Neto , L. Fu , H. Yang , Nat. Nanotechnol. 2022, 17, 378. 35115723 10.1038/s41565-021-01060-6

[49] Z. Z. Du , C. M. Wang , S. Li , H.‐Z. Lu , X. C. Xie , Nat. Commun. 2019, 10, 3047. 31296854 10.1038/s41467-019-10941-3 PMC6624286

[50] J. Zhong , S. Zhang , J. Duan , H. Peng , Q. Feng , Y. Hu , Q. Wang , J. Mao , J. Liu , Y. Yao , Nano Lett. 2024, 24, 5791. 38695400 10.1021/acs.nanolett.4c00933

[51] Y. Zhang , T.‐T. Tang , C. Girit , Z. Hao , M. C. Martin , A. Zettl , M. F. Crommie , Y. R. Shen , F. Wang , Nature 2009, 459, 820. 19516337 10.1038/nature08105

[52] L. Ma , R. Chaturvedi , P. X. Nguyen , K. Watanabe , T. Taniguchi , K. F. Mak , J. Shan , *ArXiv:2412.07150 [cond‐mat]* 2024.

