# Supplementary material for: Quantum Geometric Moment Encodes Stacking Order of Moiré Matter
Source: Adv Mater. 2025 Feb 25;37(15):2417682. doi: 10.1002/adma.202417682 (PMC12004888; doi:10.1002/adma.202417682)
Supplement: Supplementary file 1 — Supporting Information [file ADMA-37-2417682-s001.pdf]

ADVANCED MATERIALS

Supporting Information

for *Adv. Mater.*, DOI 10.1002/adma.202417682

Quantum Geometric Moment Encodes Stacking Order of Moiré Matter

Surat Layek, Subhajit Sinha, Atasi Chakraborty*, Ayshi Mukherjee, Heena Agarwal, Kenji Watanabe, Takashi Taniguchi, Amit Agarwal* and Mandar M. Deshmukh**

Supplementary Information

Quantum geometric moment encodes stacking order of moiré matter

Surat Layek[1†], Subhajit Sinha[1†*], Atasi Chakraborty[2†*], Ayshi Mukherjee[1], Heena Agarwal[1], Kenji Watanabe[3], Takashi Taniguchi[4], Amit Agarwal[5*], and Mandar M. Deshmukh[1*]

[1]*Department of Condensed Matter Physics and Materials Science, Tata Institute of Fundamental Research, Homi Bhabha Road, Mumbai 400005, India.*

[2]*Institut für Physik, Johannes Gutenberg Universität Mainz, D-55099 Mainz, Germany.*

[3]*Research Center for Functional Materials, National Institute for Materials Science, 1-1 Namiki, Tsukuba 305-0044, Japan.*

[4]*International Center for Materials Nanoarchitectonics, National Institute for Materials Science, 1-1 Namiki, Tsukuba 305-0044, Japan.*

[5]*Department of Physics, Indian Institute of Technology, Kanpur 208016, India.*

[†]These authors contributed equally to this work

[*]sinhasubhajit25@gmail.com, atasi.chakraborty@uni-mainz.de, amitag@iitk.ac.in, deshmukh@tifr.res.in

Contents

I Continuum model Hamiltonian of TDBG **3**

II Tight-Binding model of bilayer graphene **10**

II.1 Minimal TB model for AA-stacked BLG 10

II.2 Minimal TB model for AB-stacked BLG 11

II.3 Impact of Strain and generation of BCD 11

II.4 Variation of Berry curvature at band edge with perpendicular electric field 13

III Device Fabrication **14**

IV Estimation of twist angle of AB-AB and AB-BA TDBG devices **15**

IV.1 Device architecture and twist angle estimation 15

IV.2 Twist angle inhomogeneity and evidence of strain 16

V Estimation of band gap for 1.43° AB-AB TDBG device **19**

VI Additional characterization of nonlinear voltage in AB-AB and AB-BA TDBG **20**

VII Scaling Analysis and local intercept analysis **22**

VII.1 Scaling Analysis 22

VII.2 D/ϵ_0 as a parameter to probe the scaling relation 24

VII.3 Local Intercept analysis 25

VIII Additional scaling data from AB-AB TDBG devices **25**

VIII.1 Scaling data from AB-AB TDBG device-1 with twist angle 1.43° 25

VIII.2 Scaling data from AB-AB TDBG device-2 with twist angle 1.1° 27

IX Additional scaling data from AB-BA TDBG device **29**

I Continuum model Hamiltonian of TDBG

To construct the moiré Hamiltonian for different stacked TDBG platform, we first start with a brief review of the low energy model for Bernal stacked bilayer graphene building blocks. The Brillouin zone (BZ) of the AB-stacked bilayer graphene is identical to that of monolayer graphene. The primitive lattice vectors $\boldsymbol{a}_1 = a(1,0)$ and $\boldsymbol{a}_2 = a(1/2, \sqrt{3}/2)$ yield the reciprocal lattice vectors to be $\boldsymbol{b}_1^{(0)} = \frac{4\pi}{\sqrt{3}a}(\sqrt{3}/2, -1/2)$ and $\boldsymbol{b}_2^{(0)} = \frac{4\pi}{\sqrt{3}a}(0,1)$. Here, a is the lattice constant which is $\sqrt{3}$ times the carbon-carbon bond length $d = 1.42$ Å. The coordinates of the vertices of the hexagonal first BZ are $\boldsymbol{K}_\xi^{(0)} = \xi(2\boldsymbol{b}_1^{(0)} + \boldsymbol{b}_2^{(0)})/3$ with $\xi = \pm 1$ being the valley index.

Including the effects of hexagonal warping, the Hamiltonian near the K-valley can be expressed in terms of the fermion operators of the A and the B sublattice of the top and the bottom layers, $[c_A^t(k), c_B^t(k), c_A^b(k), c_B^b(k)]$, as

$$H(k) = \begin{pmatrix} h_k^t & t_k \\ t_k^\dagger & h_k^b \end{pmatrix}. \tag{1}$$

Here, the block diagonal matrices $h_k^{t/b}$ represent the 2×2 massive Dirac Hamiltonian of the top and bottom monolayers and t_k represents the effect of inter-layer hopping. The corresponding matrices are

$$h_k^{(t/b)} = \hbar v_0 \boldsymbol{\sigma}\cdot\boldsymbol{k} + \frac{\delta}{2}(\mathbb{I} \mp \sigma_z), \quad t_k = \begin{pmatrix} -\hbar v_4 \pi^\dagger & -\hbar v_3 \pi \\ \gamma_1 & -\hbar v_4 \pi^\dagger \end{pmatrix}, \tag{2}$$

with $\pi \equiv k_x + ik_y$. In the Hamiltonian, different intra-layer and inter-layer couplings have been introduced through the hopping parameter γ_i or equivalently by $v_i = \sqrt{3}|\gamma_i|a/(2\hbar)$. The nearest neighbor intra-layer coupling between the A and the B sublattice is represented by the parameter v_0. The inter-layer intra-dimer coupling is represented through γ_1. Parameters γ_3 and γ_4 are the couplings between the inter-layer non-dimer sites and the inter-layer coupling between dimer and non-dimer sites, respectively. For our calculations we consider $\delta = 15$ meV, $\gamma_0 = -3.1$ eV, $\gamma_3 = 283$ meV and $\gamma_4 = 138$ meV.

The reciprocal lattice vectors of the moiré lattice are obtained as $\boldsymbol{G}_m^i = \boldsymbol{b}_i^{(1)} - \boldsymbol{b}_i^{(2)}$, with the rotated reciprocal lattice vectors of each bilayer being specified by $\boldsymbol{b}_1^{(l)} = \mathcal{R}(\mp\theta/2)\boldsymbol{b}_1^{(0)}$ with $\mp$ for bilayer $l = 1, 2$, respectively. Using this, we obtain the pair of primitive moiré lattice vectors to be $\boldsymbol{G}_m^1 = \frac{8\pi}{\sqrt{3}a}\sin\frac{\theta}{2}(-1/2, \sqrt{3}/2)$ and $\boldsymbol{G}_m^2 = \frac{8\pi}{\sqrt{3}a}\sin\frac{\theta}{2}(1/2, \sqrt{3}/2)$. Using the low energy Hamiltonian

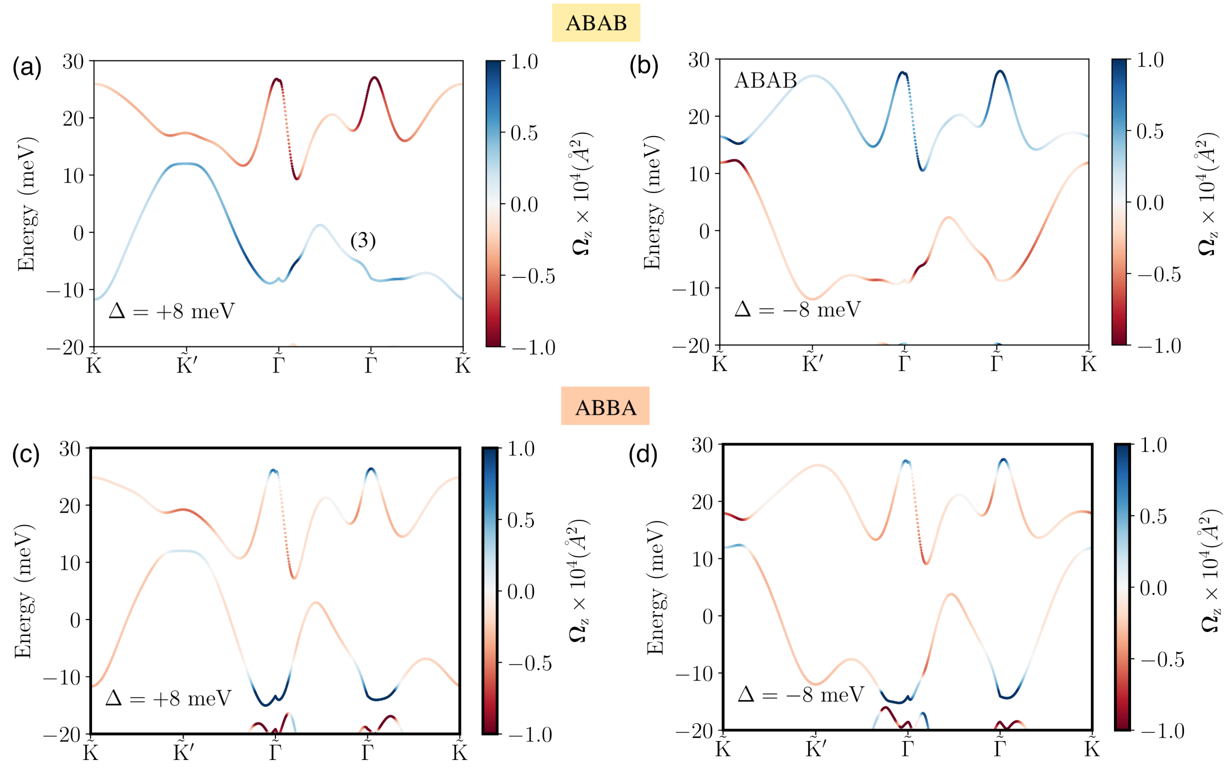

Fig. S1: Band dispersion along high-symmetry paths in presence of $\epsilon = 0.2\%$ strain. (a) and (b) represent the Berry curvature color coded energy dispersion of AB-AB stacked TDBG for $\Delta = 8$ meV and $\Delta = -8$ meV respectively. The Berry curvature for each band changes sign on reversing the direction of displacement field which is captured by the sign change of Δ. Momentum-dependent Berry curvature resolved band-dispersion of AB-BA for $\Delta = \pm 8$ meV are shown in (c) and (d). The sign of Berry curvature is invariant with the applied electric field direction for AB-BA stacked TDBG.

[Eq. (1)] for each lattice point, vertices of the small moiré hexagons, and the moiré hopping matrix, we construct the continuum Hamiltonian for TDBG. A certain cut-off in the reciprocal space is used to truncate the lattice. The smallest TDBG AB-AB Hamiltonian for the K-valley can be written as [1–4]

$$\mathcal{H} = \begin{pmatrix} h^+_{k,t} + \Delta^+_t & t^+_k & 0 & 0 \\ t^{+\dagger}_k & h^+_{k,b} + \Delta^+_b & T & 0 \\ 0 & T^\dagger & h^-_{k,t} + \Delta^-_t & t^-_k \\ 0 & 0 & t^{-\dagger}_k & h^-_{k,b} + \Delta^-_b \end{pmatrix}. \quad (3)$$

Here, the superscripts on $h^\pm_{k,t/b}$ represents rotated Dirac Hamiltonian as $h^\pm = \mathcal{R}(\mp\theta/2)\boldsymbol{k}\cdot\boldsymbol{\sigma}$ and $\Delta^\pm_{t/b}$ represents the effect of the perpendicular electric field. In Eq. (3), $T(\boldsymbol{r})$ represents the moiré coupling matrix, which connects the bottom B-layer of bilayer-1 to the top layer of A-bilayer-2.

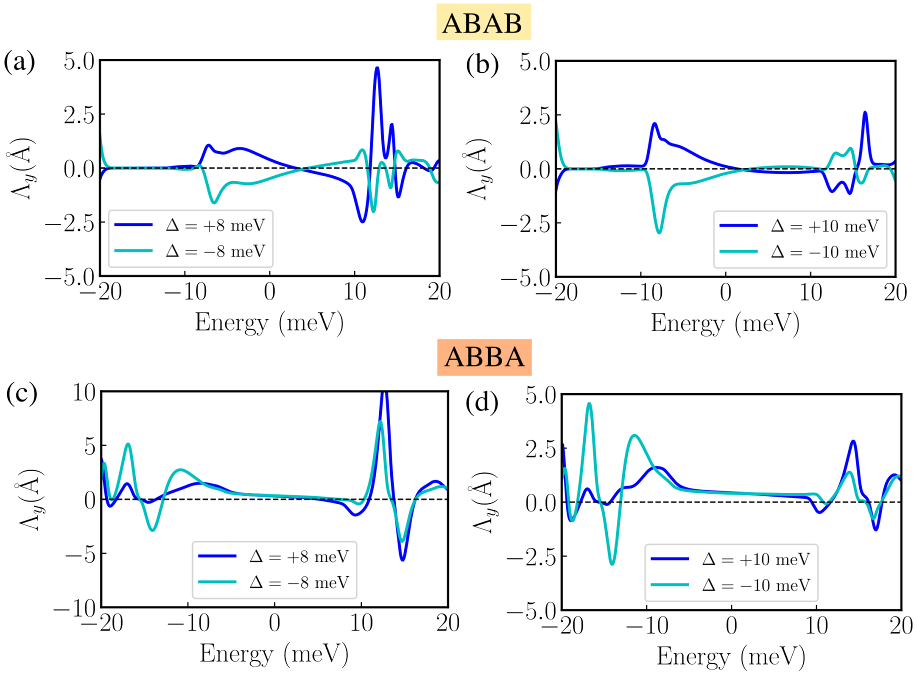

Fig. S2: Variation of Berry Curvature Dipole with the sign of electric field. The line cut of BCD with the energy of AB-AB TDBG for ±8 meV (a) and ±10 meV (b). The BCD flips sign with opposite Δ. (c) and (d) represent the BCD for ±8 meV and ±10 meV similarly for AB-BA TDBG. Here the BCD peak-structure shows the same sign for opposite out-of-plane electric field values.

For the AB-BA stacked he the double-bilayer graphene, the inter-layer hopping matrix T connects the bottom B-layer of bilayer-1 to the top layer of B-bilayer-2.

In this smallest TDBG Hamiltonian, only the nearest neighbor coupling will be considered, which is connected by the vectors $\boldsymbol{q}_b = \frac{8\pi}{3a}\sin\frac{\theta}{2}(0,-1)$ and $\boldsymbol{q}_{tl} = \frac{8\pi}{3a}\sin\frac{\theta}{2}(-\sqrt{3}/2, 1/2)$ and $\boldsymbol{q}_{tr} = \frac{8\pi}{3a}\sin\frac{\theta}{2}(\sqrt{3}/2, 1/2)$. The moiré hopping matrices are given by

$$T(\boldsymbol{r}) = \sum_{j=b,tr,tl} T_{\boldsymbol{q}_j} e^{-i\boldsymbol{q}_j\cdot\boldsymbol{r}} \,, \quad \text{where} \tag{4}$$

$$T_b = \begin{pmatrix} \omega & \omega' \\ \omega' & \omega \end{pmatrix} \qquad T_{tr/tl} = \begin{pmatrix} \omega & \omega' e^{\mp i2\pi/3} \\ \omega' e^{\mp i2\pi/3} & \omega \end{pmatrix} . \tag{5}$$

Here, ω and ω' denote the diagonal and the off-diagonal hopping strengths, respectively. We emphasize that an unequal ω and ω', specifically $\omega' > \omega$, is crucial to match the calculated low energy band structure with the experimentally observed spectral gap [5, 6]. In this paper, we consider $\omega' = 106$ meV and $\omega = 79$ meV [5, 7].

In the presence of uni-axial strain ($\mathcal{E}$), the Dirac Hamiltonian of Eq. (3) modifies to

$$h_{k,l} = \hbar v_0 \mathcal{R}(\mp\theta/2)\ [(\mathbb{I} + \mathcal{E}^T)](\boldsymbol{k} - \boldsymbol{D}_\xi) \cdot (\xi\sigma_x, \sigma_y) + \frac{\delta}{2}(\mathbb{I} \mp \sigma_z)\ . \tag{6}$$

The complete derivation of strain-induced modifications is provided in Ref. [1, 3, 8]. The strain matrix operates over the position of the twisted Dirac points given by

$$\boldsymbol{D}_\xi = (\mathbb{I} - \mathcal{E}^T)\boldsymbol{K}^i_\xi - \xi\boldsymbol{A}\ , \tag{7}$$

with $\boldsymbol{A}$ representing the gauge field that has the dimension of the reciprocal lattice vector. The appearance of the gauge field can be attributed to the fact that the strain causes the inter-atomic distance in each layer to become different in different directions. This results in the difference of hopping parameters, which displaces the Dirac point from its original position. The gauge potential $\boldsymbol{A}$ in terms of the strain matrix elements is given by

$$\boldsymbol{A} = \frac{\sqrt{3}}{2a}\beta(\mathcal{E}_{xx} - \mathcal{E}_{yy}, -2\mathcal{E}_{xy})\ . \tag{8}$$

Here, $\beta = 1.57$ and $\mathcal{E}_{ij}$ are the elements of the strain matrix. Strain also modifies the lattice vectors and consequently, the hopping matrices and the hopping vectors. We calculate the strained moiré vectors starting from un-rotated and un-strained lattice vectors. We obtain the lattice vectors for the strained lattice using $\boldsymbol{G}_m^{1,\mathrm{st}} = R_{-\frac{\theta}{2}}(1 - \mathcal{E}^T)\boldsymbol{b}_1 - R_{\frac{\theta}{2}}\boldsymbol{b}_1$ and $\boldsymbol{G}_m^{2,\mathrm{st}} = R_{-\frac{\theta}{2}}(1 - \mathcal{E}^T)\boldsymbol{b}_2 - R_{\frac{\theta}{2}}\boldsymbol{b}_2$.

The band dispersions for the lowest energy valence and conduction bands in ABAB and ABBA stacking are shown in Fig. S1 (a, b) and (c, d) for $\Delta = \pm 8$ eV in the presence of 0.2% strain. Next, we calculate the Berry curvature (BC) which is defined as $\Omega^n_d = \frac{1}{2}\epsilon_{dac}\Omega^n_{ac}$, where

$$\Omega^n_{ac} = -2\mathrm{Im}\sum_{m\neq n}\frac{\langle u_n|\partial_{k_a}\mathcal{H}|u_m\rangle\langle u_m|\partial_{k_c}\mathcal{H}|u_n\rangle}{(\epsilon_n - \epsilon_m)^2}\ . \tag{9}$$

Here, u_n is the periodic part of the Bloch wave-function where $\mathcal{H}|u_n\rangle = \epsilon_n|u_n\rangle$ corresponds to the moiré bands. In ABAB-stacked TDBG, the BC hotspot at the band edge reverses sign with the switching of electric field direction. In contrast, the sign of BC and the associated valley Chern number, $Z_2 \equiv (C_K - C_{K'})/2$ in ABBA-stacked TDBG remain unaffected by the electric field

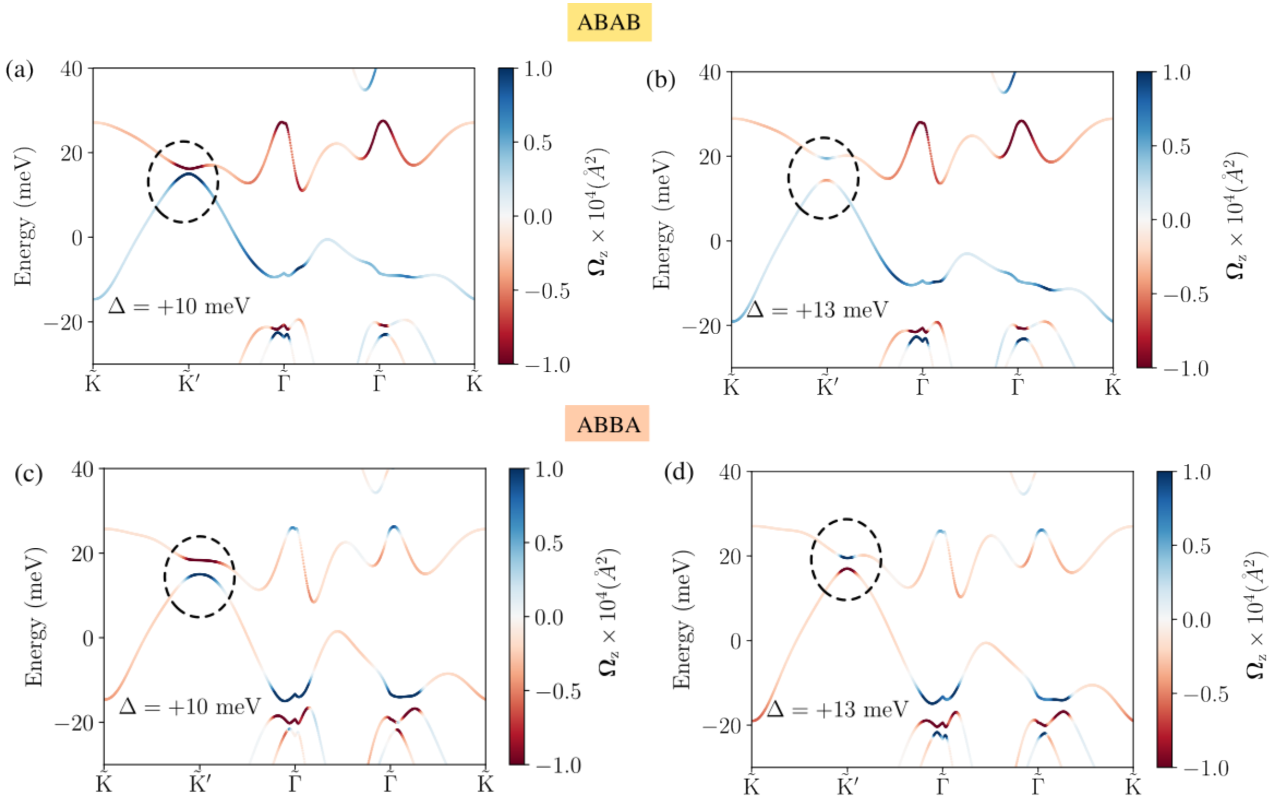

Fig. S3: Band dispersion around topological phase transition in presence of $\epsilon = 0.2\%$ strain. (a) and (b) represent the Berry curvature color-coded energy dispersion of AB-AB stacked TDBG for $\Delta = 10$ meV and $\Delta = 13$ meV respectively. The gap closing between CB and VB around $\Delta = 11$ meV captures the topological transition. The Berry curvature changes sign across the two phases of phase transiton. Momentum dependent Berry curvature resolved band-dispersion of AB-BA for $\Delta = 10$ meV and $\Delta = 13$ meV are shown in (c) and (d). The sign change across topological transition is invariant with respect to stacking order.

polarity. Here, C_K and $C_{K'}$ denote Chern numbers of the individual bands at $\mathbf{K}$ and $\mathbf{K'}$ valleys, respectively.

After analyzing the electronic band structure and the BC evolution in TDBG under varying electric fields, we now focus on the second-order non-linear Hall response of the ABAB and ABBA stacked TDBG. The non-linear conductivity σ_{abc} which links the non-linear current to the in-plane electric field, $\boldsymbol{E}$ via the relation $j_a^{(2)} = \sigma_{abc} E_b E_c$, where a, b and c represent spatial indices, is defined as

$$\sigma_{abc} = \epsilon_{abd} \frac{e^3 \tau}{\hbar^2} \Lambda^{\sigma}_{dc} \quad \text{where,} \;\; \Lambda^{\sigma}_{dc} = -\sum_n \int [d\boldsymbol{k}] \frac{\partial f_0^n}{\partial k_c} \Omega_d^n \; . \tag{10}$$

Here, $-e$ is the electronic charge, ϵ_{abd} is the anti-symmetric Levi-Civita tensor, τ is the scattering time and Λ^{σ}_{dc} is the BCD.

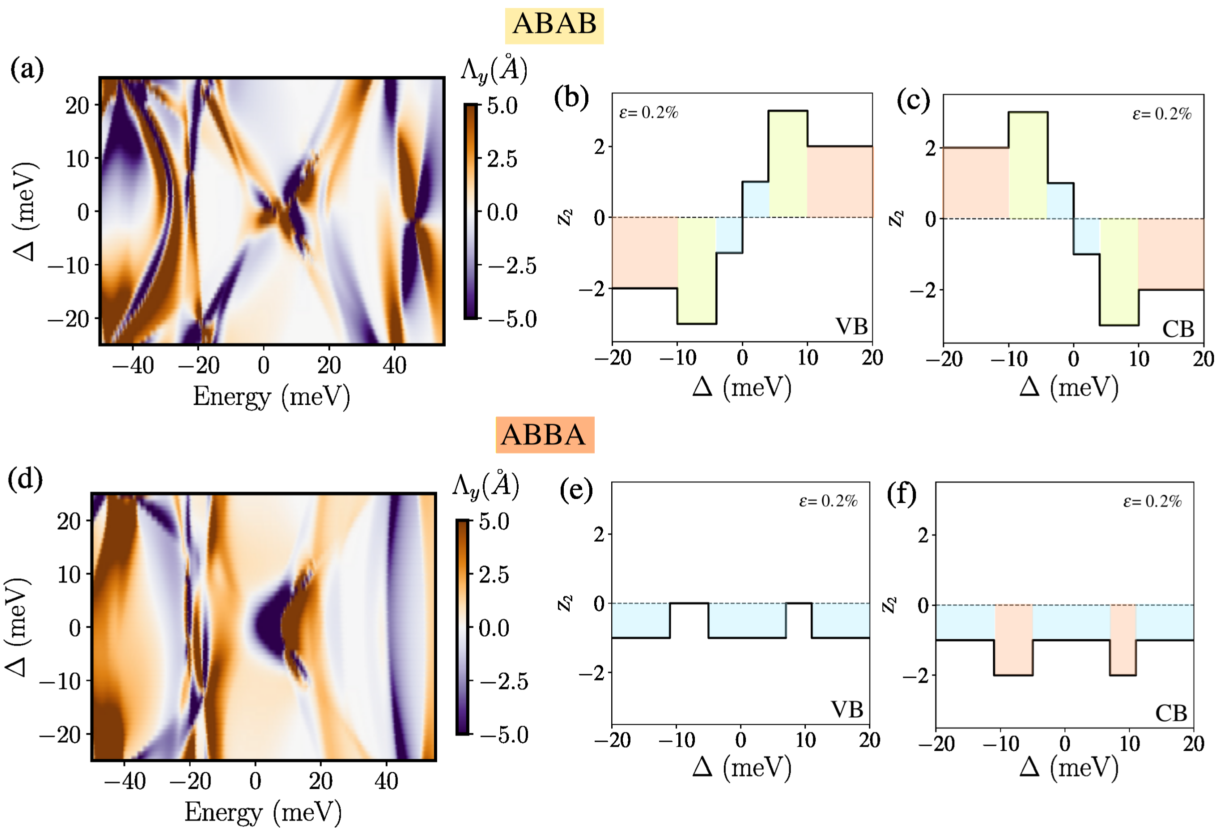

Fig. S4: BCD phase diagram and Chern number. Variation of BCD over $\Delta-$Energy plane (a) for AB-AB, (d) for AB-BA in presence of strain $\epsilon = 0.2\%$. The change in the valley chern number Z_2 of ABAB-TDBG for lowest valence band (VB) (b) and conduction band (CB) (c) for fixed strain $\epsilon = 0.2\%$. The Chern number changes sign between positive and negative Δ values for both VB and CB. Similarly, the valley Chern numbers of VB and CB for $\epsilon = 0.2\%$ are plotted in (e) and (f) for ABBA-TDBG. The valley Chern number is symmetric with respect to the change is the direction of electric field for ABBA-stacked TDBG.

In Fig. S2 (a,b) and (c,d) we plot the variation of λ_y with energy near the charge neutrality for ABAB and ABBA stacking, considering two different values of the inter-layer potential Δ =8 eV and 10 eV. Notably, for both the field strengths, reversing the electric field direction, as indicated by the sign of Δ, results in a sign reversal of BCD in ABAB-stacked TDBG. However, for ABBA-stacked TDBG, the BCD sign remains unaffected by changes in the field direction. The slight variation in the response magnitude between positive and negative Δ likely arises from the influence of strain, and other changes in the band structure.

Remarkably, the variation of the perpendicular electric field in both ABAB and ABBA-stacked TDBG can induce topological phase-transition of the valley-Chern type. Similar to the usual phase-transition in Chern insulators, the valley-Chern number changes in TDBG are also associated with the band gap closing at specific k point. To illustrate this, we explicitly examined the evolution

of the band movement in Fig. S3 in presence of $\epsilon = 0.2\%$ strain. The BC swaps between the band-touching edges between the consecutive bands across the transition at $\Delta = 12$ meV as we see in Fig. S3 (a,b) for ABAB and similarly in Fig. S3 (c,d) for ABBA. We find multiple phase transitions in both ABAB- and ABBA-stacked TDBG on varying electric field. The distribution of λ_y is plotted over the $\Delta-$Energy plane in Fig. S4 (a, d).

To track the changes in the band topology, we plot the valley Chern number Z_2 for ABAB stacked TDBG in Fig. S4 (b), (c) for the lowest valence and conduction band for a fixed strain strength of $\epsilon = 0.2\%$. We find three topological phase transitions within range Δ =0 to 20 meV. Interestingly, the valley Chern number switches sign with the sign change of Δ. In contrast, the Chern histogram shown in Fig. S4 (e, f) for ABBA VB and CB exhibit a symmetric nature with respect to positive and negative Δ. This captures the distinct topological phases induced by the stacking order, which exhibit different behavior on reversing the polarity of the vertical electric field. We show below that this imprint of stacking order on band topology can be captured by the non-linear transport experiments.

While our theoretical model provides qualitative insights consistent with experimental conclusions, quantitative differences may arise due to the sensitivity of calculated BCD to strain direction (e.g., Fig. 4 of Pantaleon et al. [9]) and magnitude (Section V of Supplementary Information in Sinha et al. [1]). We used a simplified uniaxial strain model along the zigzag direction of graphene, whereas an experimental strain is likely more complex and uncontrolled in magnitude or orientation. This can be one of the primary causes for the difference between the BCD values calculated in theory and obtained in experiments. For this reason, we do not focus on the magnitude of BCD, but use the BCD sign changes with the polarity of the perpendicular electric field to decipher the TDBG stacking order. Furthermore, the exact values of used parameters to calculate the band structure in twisted graphene systems are still being debated in the literature, even though they capture the essential physics. Despite these limitations, our theoretical framework effectively explains the contrasting BCD behavior observed in AB-AB and AB-BA stacked TDBG.

II Tight-Binding model of bilayer graphene

To understand the stacking-mediated band-topology, in this section, we focus on the simple bilayer graphene (BLG) model to calculate the electronic bands, their BC and first moment, BCD. In the following, we introduce 4×4 low energy tight binding (TB) model Hamiltonian for AA- and AB-stacked BLG model [10–12].

II.1 Minimal TB model for AA-stacked BLG

The low energy Hamiltonian for AA-stacked bilayer graphene (BLG) in the basis $[l_A^1,\ l_B^1,\ l_A^2,\ l_B^2]$ is expressed as,

$$\begin{pmatrix} -\Delta & -\gamma_0\phi(\mathbf{k}) & \gamma_1 & 0 \\ -\gamma_0\phi^*(\mathbf{k}) & -\Delta & 0 & \gamma_1 \\ \gamma_1 & 0 & +\Delta & -\gamma_0\phi(\mathbf{k}) \\ 0 & \gamma_1 & -\gamma_0\phi^*(\mathbf{k}) & +\Delta \end{pmatrix},$$

where l_α^i represent the α sublattice of layer i. The geometric factor is given by $\phi(\mathbf{k}) = \sum_{l=1}^{3} e^{i\mathbf{k}\cdot\delta_\mathbf{l}}$ where $\delta_\mathbf{l}$ denotes the positions of the three nearest B sublattice relative to the A sublattice or vice-versa within one monolayer graphene. The three connecting vectors can be expressed as $\delta_\mathbf{1} = (\mathbf{a}_1 - \mathbf{a}_2)/3$, $\delta_\mathbf{2} = (\mathbf{a}_1 + 2\mathbf{a}_2)/3$, and $\delta_\mathbf{3} = -(2\mathbf{a}_1 + \mathbf{a}_2)/3$. Here γ_0 and γ_1 are intra-layer and inter-layer hopping elements. We used the following model parameters $\gamma_0 = 3.16$ eV and $\gamma_1 = 0.381$ eV for our calculation. We disregard the negligible inter-layer hopping between non-dimeric sites in the model. The layer-dependent potential, Δ, is used to account for the effect of the perpendicular electric field.

The sole presence of an electric field alone is not sufficient to open a gap for AA-stacked BLG. Note that, the existence of a band gap is crucial for estimating the Berry curvature. To address this, we introduced a small artificial corrugation effect by incorporating a perturbative H^{cor} Hamiltonian to the original Hamiltonian. Here we set, $H_{11}^{\text{cor}} = \delta_{cor}$, $H_{22}^{\text{cor}} = H_{33}^{\text{cor}} = H_{44}^{\text{cor}} = -\delta_{cor}$ and $H_{ij}^{\text{cor}} = 0$ for $i \neq j$ for the calculation of energy dispersion. δ_{cor} represent the artifical corrugation gap. The energy dispersion of the artificially corrugated AA-stacked BLG with δ_{cor} =0.08 eV for $\Delta = \pm 0.1$

eV is included in Fig. 5 of the main manuscript.

II.2 Minimal TB model for AB-stacked BLG

In this section, we present the low energy minimal Hamiltonian for AB-stacked namely Bernal BLG in the $[l^1_A,\ l^1_B,\ l^2_A,\ l^2_B]$ basis. The Hamiltonian, accounting for the effect of the electric field, has the following expression,

$$\begin{pmatrix} -\Delta & -\gamma_0\phi(\mathbf{k}) & 0 & 0 \\ -\gamma_0\phi^*(\mathbf{k}) & -\Delta & \gamma_1 & 0 \\ 0 & \gamma_1 & +\Delta & -\gamma_0\phi(\mathbf{k}) \\ 0 & 0 & -\gamma_0\phi^*(\mathbf{k}) & +\Delta \end{pmatrix},$$

The incorporation of the perpendicular electric field Δ opens up a gap between the valence and conduction band, breaking the inversion symmetry of the AB-BLG. The energy dispersion of the pristine AB-stacked BLG for $\Delta = \pm 0.1$ eV are included in Fig. 5 of the main manuscript.

II.3 Impact of Strain and generation of BCD

The breakdown of inversion symmetry is essential to have a finite Berry curvature in systems preserving time-reversal symmetry. However, pristine BLG has three-fold rotational symmetry (C3) that forbids BCD. Therefore, we apply a uniaxial strain in the graphene disrupting the C3 symmetry to overcome this limitation. Consider a uniaxial strain of strength ϵ, applied along an arbitrary angle ϕ relative to the zig-zag direction. It can be described by the following strain tensor

$$\mathcal{E} = \epsilon \begin{pmatrix} -\cos^2\phi + \nu\sin^2\phi & -(1+\nu)\sin\phi\cos\phi \\ -(1+\nu)\sin\phi\cos\phi & -\sin^2\phi + \nu\cos^2\phi \end{pmatrix}, \tag{11}$$

Here, ϵ is the strength of the strain, ν is the Poisson ratio (~ 0.16 for graphene) and ϕ is the strain angle w.r.t zigzag direction of graphene. The applied uniaxial strain distorts the lattice structure with $\mathbf{a} \to \mathbf{a}' = (\mathbb{1} + \mathcal{E})\mathbf{a}$ and renormalizes reciprocal lattice vectors with $\mathbf{b} \to \mathbf{b}' = (\mathbb{1} - \mathcal{E}^T)\mathbf{b}$. The corresponding hopping parameters modifies to $\gamma_0 \to \gamma_0 e^{-\beta(\frac{|\delta'|}{|a_0|}-1)}$, where $\beta = 1.57$. Here $\mathbf{a_0} \equiv \frac{\mathbf{a}}{\sqrt{3}}$

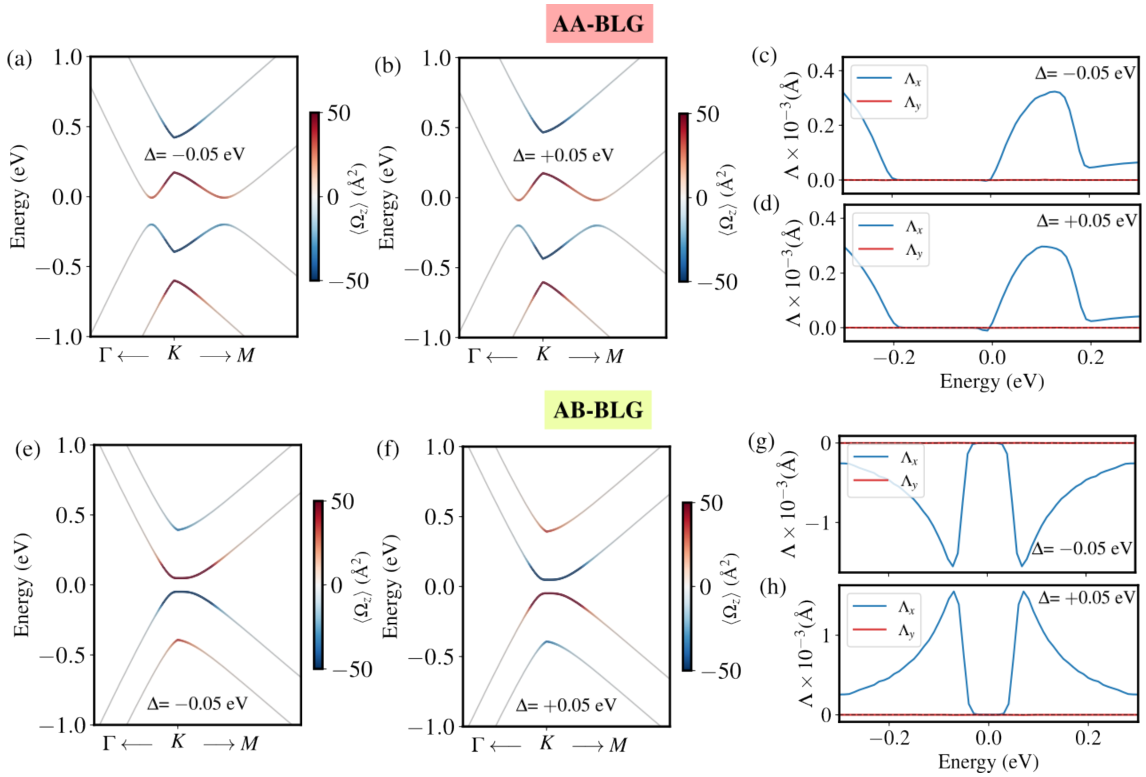

Fig. S5: Band dispersion and Berry curvature dipole of strained BLG. (a) and (b) show the strained energy dispersion of artificial corrugated AA-stacked BLG for $\Delta = -0.05$ eV and 0.05 eV respectively. We choose strain $\epsilon = 1.0\%$ for our plot. The corresponding BCD over the energy for $\Delta = \mp 0.05$ are plotted in (c) and (d). The strained band-structures around high-symmetry $\mathbf{K}$ point are shown in (e) and (f) for $\Delta = \mp 0.05$. The corresponding BCD of AB-stacked BLG over the energy for $\Delta = \mp 0.05$ are plotted in (g) and (h). The BCD of AB-BLG changes sign with direction flip of the electric field.

is the bond length between adjacent carbon atoms of monolayer graphene, and we choose $\phi = 0$, i.e. uniaxial strain along the zigzag direction of the graphene layer.

The energy dispersions of the AA and AB stacked BLG in the presence of $\epsilon = 1\%$ strain are plotted in Fig. S5. We choose $\Delta = -0.05$ eV along with the artificial corrugation potential $\delta_{cor} = 0.2$ eV to calculate the band dispersion of AA stacked BLG as shown in Fig. S5 (a). The energy dispersion around high symmetry $\mathbf{K}-$point for AB stacked BLG is shown in Fig. S5 (c). Clearly, the BC hot spot lies at the band edge. For both stacking, the lowest conduction and valence band pairs host opposite BC. Next, we change the sign of the Δ to account the change of direction of perpendicular electric field and plot the band dispersion in Fig. S5 (b) and (f) for AA- and AB-stacked BLG. The switching of the electric field, changes the sign of BC for AB-BLG whereas the nature of BC remains same for AA-BLG.

To investigate the variation of BCD with change of electric field direction we plot Λ_x and Λ_y

for $\Delta = \mp 0.05$ for AA-stacked corrugated BLG (see Fig. S5 c, d) and Bernal AB-stacked BLG (see Fig. S5 g, h). The BCD of AA-stacked BLG remains unaffected by the sign change of the electric field. However, the BCD changes sign with electric field direction for AB-stacked BLG. Hence, the BCD variation on reversing the direction of the electric field is sensitive to the stacking order.

II.4 Variation of Berry curvature at band edge with perpendicular electric field

In this section, we explore the change in the magnitude of the BC hot-spot with perpendicular electric field. We systematically increased the Δ from 0.01 eV to 0.15 eV for Bernal AB-stacked BLG. The change in the direct band-gap at the charge-neutrality with variation of Δ is plotted in Fig. S6 (a). The band-gap increases almost linearly with the increment of perpendicular electric field Δ. We plot the magnitude of BC at valence band-edge for the corresponding Δ range. Variation of Ω_z at VB w.r.t Δ is shown in Fig. S6 (b). Interestingly, the BC changes sharply for the low Δ values. The rate of change in BC decreases for the larger values of Δ which is consistent with the findings of Ref. [13]. For this reason, we choose high Δ range for the fitting of the experimental data.

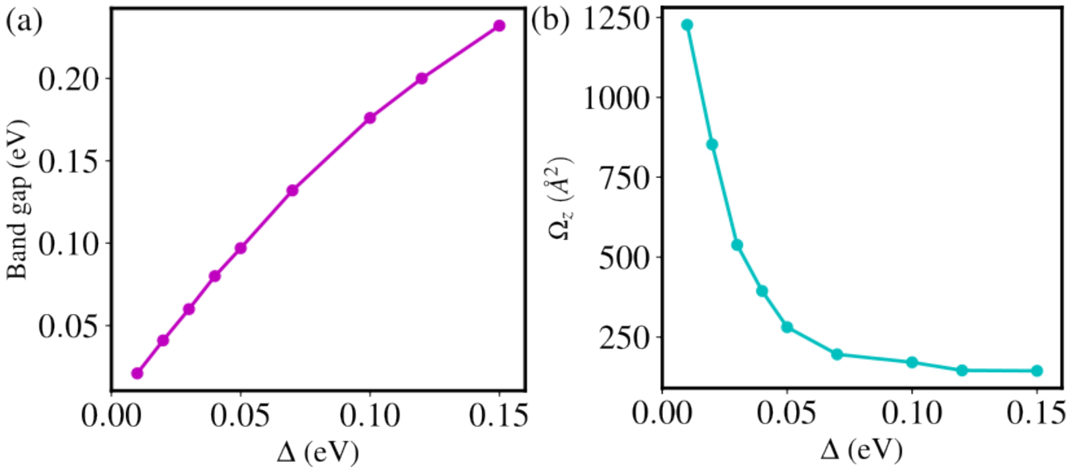

Fig. S6: Variation of band-gap and BC with electric field of BLG. (a) The magnitude of direct band gap between the lowest valence and conduction band of AB-stacked BLG for different values of Δ. (b) shows the value of BC at valence band edge with variation of Δ. The BC falls sharply for low Δ values. For the higher value of perpendicular electric field, the rate of change of BC is relatively small.

III Device Fabrication

ABAB-TDBG and ABBA-TDBG samples were fabricated using the cut-and-stack method. Bilayer graphene and h-BN flakes (thickness: 20-40 nm) were exfoliated onto a 285 nm thick SiO_2/Si++ substrates. A Bernal-stacked (AB) bilayer graphene flake was first cut into two pieces using a tapered optical fiber scalpel [14] to ensure that both pieces shared the same crystallographic axis.

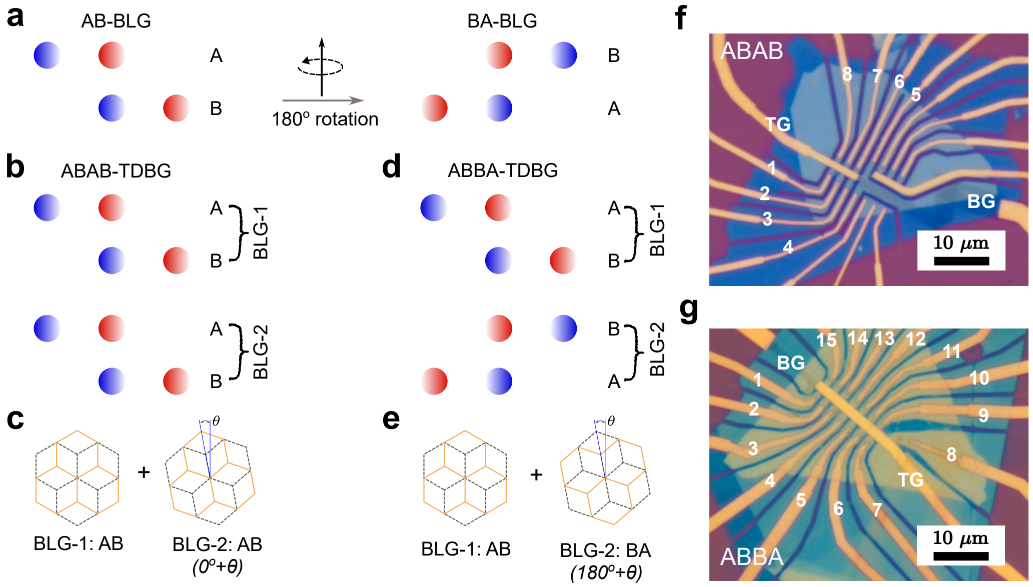

Fig. S7: Fabrication of AB-AB and AB-BA TDBG devices. (a) Atomic arrangements in AB-stacked bilayer graphene (AB-BLG) and its transformation into BA-stacked bilayer graphene (BA-BLG) upon a 180° rotation. Red and blue spheres represent the two distinct sublattices of graphene. (b) Two AB-stacked bilayer graphene (BLG-1 and BLG-2) layers are stacked on top of each other, forming the AB-AB TDBG. (c) Two AB-BLGs are twisted relative to each other by an angle θ, resulting in the twisted AB-AB TDBG structure. The dashed and solid lines indicate the top and bottom layers within a single BLG. (d) The AB-stacked BLG-1 and BA-stacked BLG-2, created by an additional 180° rotation of AB-BLG, are stacked together to form AB-BA TDBG. (e) Two BLGs are twisted relative to each other by $180^\circ + \theta$, forming the twisted AB-BA TDBG structure. (f) Optical image of the fabricated AB-AB TDBG device with a relative twist angle of 1.43°. Top and bottom gates (TG and BG) and numbered contact electrodes are visible. The width and the length of the region used for measurements are 1 μm and 1.75 μm respectively. (g) Optical image of the fabricated AB-BA TDBG device with a relative twist angle of 1.4°. Top and bottom gates (TG and BG) and numbered contact electrodes are labeled. Scale bars represent 10 μm. The width and the length of the region used for measurements are 1.5 μm and 1.95 μm respectively.

To assemble the heterostructures, an h-BN flake, serving as the dielectric layer for the top gate, was picked up using a PC (Poly (Bisphenol A carbonate)) + PDMS (polydimethylsiloxane) stamp at 110 °C. One half of the bilayer graphene was then picked up at 90 °C using this h-BN flake. The second half was subsequently picked up with a rotation angle of θ ($180^\circ+\theta$) relative to the first half, forming either the ABAB-TDBG (ABBA-TDBG) configuration. The rationale for the 180° rotation is schematically depicted in Fig. S7. Following this, an additional h-BN flake, serving as

the dielectric layer for the bottom gate, and a few-layer graphite flake were sequentially picked up at 90-100 °C.

The stack was transferred onto Si++/SiO_2 substrates that had been treated with O_2 reactive ion etching (40 sccm O_2, power: 25 W, pressure: 1 Pa) to prepare the surface. Residual PC was removed by rinsing the samples in chloroform. The heterostructures were then patterned into dual-gate Hall bar devices using electron beam lithography. Cr/Au (5 nm/60 nm) was deposited to form the top gate electrode, while a few-layer graphite was used as the back-gate electrode.

To establish edge contacts, the top h-BN layer was etched using CHF_3/O_2 plasma (CHF_3: 40 sccm, O_2: 4 sccm, power: 60 W, pressure: 1 Pa). Finally, Cr/Pd/Au (5 nm/20 nm/40 nm) contacts were deposited using electron beam evaporation, following in situ Ar plasma cleaning. A non-zero fixed global gate voltage was applied to the heavily doped silicon to further reduce the contact resistances. Ohmic behavior of the two probe contact resistances were verified.

IV Estimation of twist angle of AB-AB and AB-BA TDBG devices

IV.1 Device architecture and twist angle estimation

We performed low-temperature transport measurements primarily at 1.2 K-1.5 K, unless otherwise specified, for the AB-AB and AB-BA TDBG devices, using a He_4 flow cryostat. A current of 100 nA with a frequency (ω) of 177 Hz was applied and the four-probe longitudinal resistance $\mathrm{R}_{xx} = \mathrm{V}^{\omega}_{xx}/I_{\omega}$ was measured using SR-830 lock-in amplifier, following amplification using a DL instrument voltage preamplifier.

The charge density (n) and the perpendicular electric displacement field (D) were determined using the formulas $n = (C_{\mathrm{BG}}V_{\mathrm{BG}} + C_{\mathrm{TG}}V_{\mathrm{TG}})/e - n_0$ and $D = (C_{\mathrm{BG}}V_{\mathrm{BG}} - C_{\mathrm{TG}}V_{\mathrm{TG}})/2$. Here, C_{TG} and C_{BG} represent the capacitance per unit area of the top and back gates, respectively, e denotes the electron charge, and n_0 is the offset in charge density due to unintentional doping. V_{BG} and V_{TG} denote the DC voltage applied to the back gate and top gate, respectively. The capacitance values were derived using the hBN dielectric thickness of a gate and the slope of R_{xx} peak at the charge neutrality in the V_{BG}-V_{TG} plane. The values were subsequently verified via analysis of

magneto-transport features, such as the positions of Brown-Zak oscillations (see Fig. S8b) and the tracing of Landau levels (see Fig. S8b and Fig. S9b) from the fan diagram.

Figure S8a shows the measured R_{xx} in the full parameter space of n and perpendicular electric field D/ϵ_0. The fan diagrams at $D/\epsilon_0 = 0$ for AB-AB (AB-BA), displayed in Fig. S8b (Fig. S9b), were obtained with magnetic fields reaching up to 13.6 T. The overlaid lines on the fan diagram, originating from $\nu = 0$ and ± 4, correspond to different Landau levels. The horizontal grey dashed lines in Fig. S8b correspond to Brown-Zak oscillations, resulting in R_{xx} dips visible most clearly on the (-12,0) line for the AB-AB device.

To determine the twist angle (θ), we estimated the value of n_S (see Fig. S8c for AB-AB device-1 and Fig. S9a for AB-BA device used in the main manuscript) from our low-temperature electron transport measurements. The twist angle is then extracted utilizing the relation $n_S = 8\theta^2/\sqrt{3}a^2$. In this equation, n_S denotes the charge carrier density corresponding to the full filling of the moirè band ($\nu = \pm 4$), and $a = 0.246$ nm is the lattice constant of graphene. The AB-AB TDBG device-2 with an angle of 1.1°, used in Section VIII.2, has been characterized similarly [15].

IV.2 Twist angle inhomogeneity and evidence of strain

Strain is a crucial parameter in twisted graphene devices, as it breaks the C_3 symmetry [16, 17] and induces a finite Berry curvature dipole. Evidence of strain in our TDBG devices is demonstrated by the presence of angle inhomogeneity. Specifically, if two different predominant angles exist within our device, they correspond to two distinct peaks in R_{xx} vs. n dependence at full-filling ($n = \pm n_S$). Our transport measurements revealed two sub-peaks at the moirè peak (see Fig. S8d), indicating two different n_S values. From this observation, we estimated $n_{S1} = 4.73 \times 10^{12}$ cm^{-2} and $n_{S2} = 4.80 \times 10^{12}$ cm^{-2}, which correspond to twist angles $\theta_1 = 1.42°$ and $\theta_2 = 1.43°$, respectively, in the AB-AB TDBG device-1 used in the main manuscript. Given that $\Delta\lambda/\lambda = \Delta\theta/\theta$, λ being the moiré wavelength, the strain in our AB-AB device is estimated to be, $(\theta_2 - \theta_1)/\theta_{avg} = .007$, where $\theta_{avg} = (\theta_1 + \theta_2)/2$.

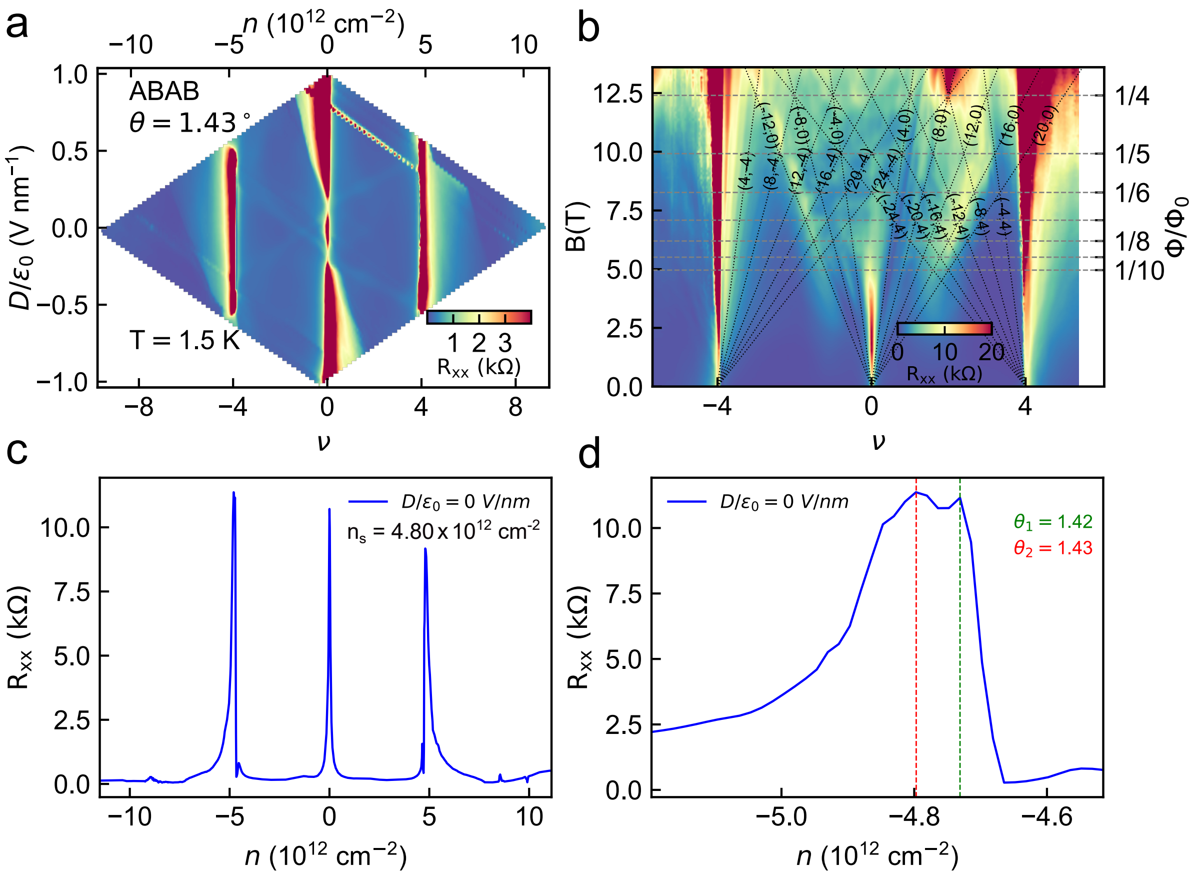

Fig. S8: Estimation of twist angle and signature of strain in AB-AB TDBG device-1. a, Four-probe longitudinal resistance R_{xx} of AB-AB TDBG device-1 (shown in Fig. 2a of the main manuscript) as a function of filling factor (ν) and perpendicular electric field (D/ϵ_0) for extended ν and D/ϵ_0 range. The top axis indicates the charge density (n). **b,** Colorscale plot of R_{xx} as a function of ν and perpendicular magnetic field B. The (ν_{LL}, ν) values indicate the Landau level filling factor ν_{LL} that originates from the filling ν at $B = 0$, and the corresponding dotted lines coincide with local resistance minima. The right axis shows the corresponding values of ϕ/ϕ_0, where ϕ is the magnetic flux through a moiré unit cell and ϕ_0 is the magnetic flux quantum. The horizontal dashed lines correspond to a decrease in R_{xx} due to Brown-Zak oscillations that occur at simple fractions of ϕ/ϕ_0. **c, d** R_{xx} vs n lineslice at D/ϵ_0= 0 V nm^{-1} for the full n range measured (c), and zoomed in close to $n = -n_S$ (d). The two subpeaks correspond to two slightly different twist angles due to strain in the sample.

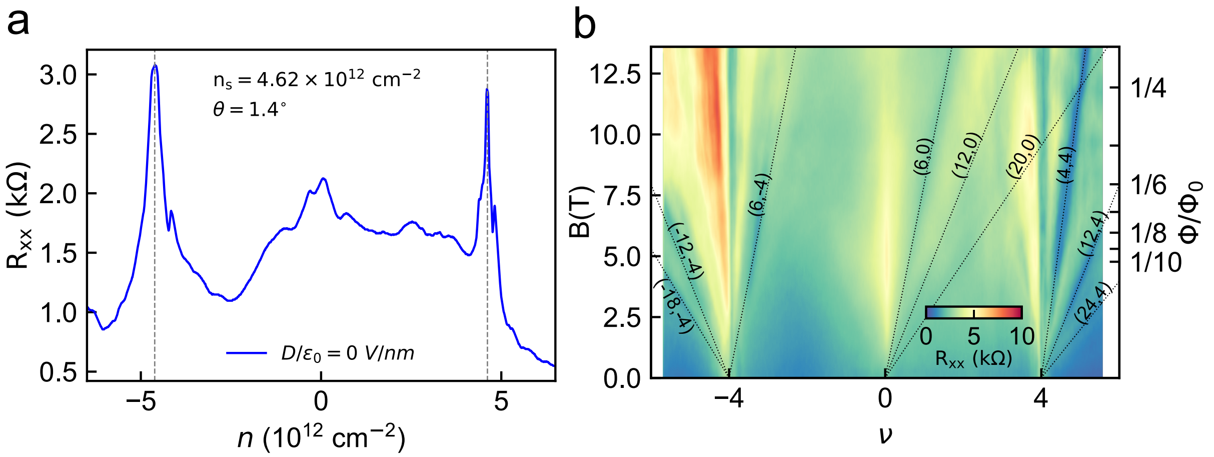

Fig. S9: Estimation of twist angle in AB-BA TDBG device. a, R_{xx} vs n lineslice at $D/\epsilon_0 = 0$ V nm^{-1} for the full n range measured at $T = 1.5$ K. The extracted value of n_S indicates a twist angle of $\theta = 1.4°$. **b,** Colorscale plot of R_{xx} as a function of ν and perpendicular magnetic field B, measured at $T = 300mK$. The ($\nu_{\rm LL}$, ν) values indicate the Landau level filling factor $\nu_{\rm LL}$ that originates from the filling ν at $B = 0$, and the corresponding dotted lines coincide with a resistance minima. The right axis shows the corresponding values of ϕ/ϕ_0, where ϕ is the magnetic flux through a moiré unit cell and ϕ_0 is the magnetic flux quantum.

V Estimation of band gap for 1.43° AB-AB TDBG device

We employed Arrhenius fitting to estimate the band gap of AB-AB device-1 and study its evolution under a perpendicular electric field at the CNP. R_{xx} vs. D/ϵ_0 dependence is measured by varying temperature upto ≈80 K (Fig. S10a). A linear fitting of the natural logarithm $\ln(R_{xx})$ with the inverse of the temperature (T^{-1}) is done, where the slope is directly related to the band gap. Figure S10c (Figure S10d) shows the linear fit in grey dashed lines for different negative (positive) values of D/ϵ_0. The Arrhenius equation, $R_{xx}(T) \propto e^{E_g/2k_BT}$, where E_g is the band gap and k_B is the Boltzmann constant, allows us to extract the band gap from the temperature-dependent resistance data.

Our observations reveal that the band gap of 1.43° AB-AB TDBG evolves with the applied perpendicular electric field at the CNP (Fig. S10b). As we varied the displacement field, we noticed the band gap initially decreased, leading to a band touching, and then reopened at a displacement field of approximately $D/\epsilon_0 \approx \pm 0.17$ V nm^{-1}.

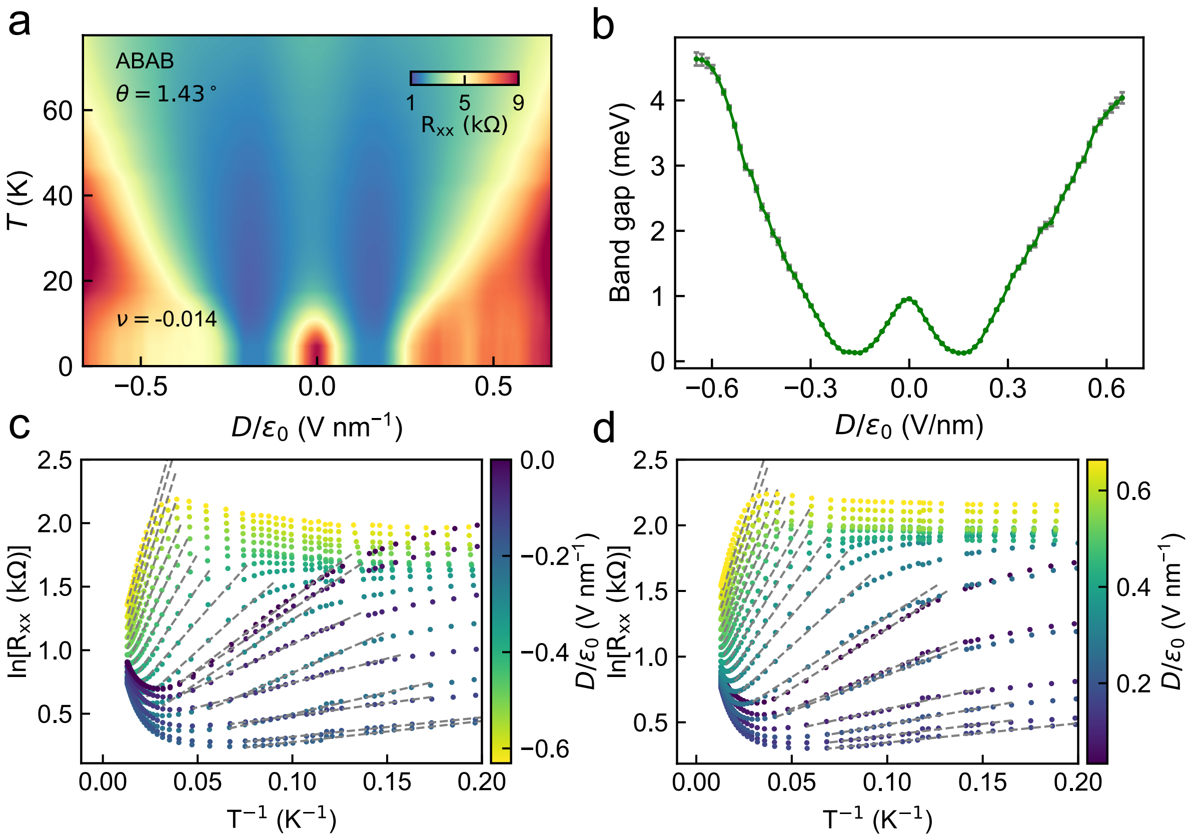

Fig. S10: Bandgap estimation near charge neutrality point for AB-AB TDBG device-1. a, Longitudinal resistance R_{xx} as a function of D/ϵ_0 and temperature T. **b,** The extracted band gap as a function of D/ϵ_0. The vertical gray bars represent the errors in bandgap extraction. **c, d,** Arrhenius band gap extraction by fitting the linear regime (dashed gray lines) in ln (R_{xx}) vs $1/T$ for $D < 0$ (c) and $D > 0$ (d). The color of data points indicates the corresponding value of D/ϵ_0 for a particular ln (R_{xx}) vs $1/T$ plot.

VI Additional characterization of nonlinear voltage in AB-AB and AB-BA TDBG

The nonlinear Hall voltage $V_{xy}^{2\omega}$ exhibits a quadratic dependence on the applied current (I_ω) having frequency ω. This quadratic dependence arises because the nonlinear Hall effect is a second-order response to the external in-plane electric field (E_{xx}^{ω}), making it sensitive to both the magnitude and direction of the applied current. Specifically, the nonlinear Hall voltage can be described by $V_{xy}^{2\omega} \propto (E_{xx}^{\omega})^2 \propto (I_\omega)^2$, indicating that as the current increases, the nonlinear Hall voltage grows quadratically. The linear longitudinal voltage V_{xx}^{ω} is directly proportional to the current, following Ohms law, $V_{xx}^{\omega} = R_{xx} I_\omega$, where R_{xx} is the longitudinal resistance. Hence, as the current is increased, $V_{xy}^{2\omega}$ changes linearly with $(V_{xx}^{\omega})^2$. In Fig. S11a, we have demonstrated the quadratic dependence

of the nonlinear Hall voltage on current up to 190 nA for the 1.43° AB-AB device. Figure S12a illustrates the linear dependence of $V_{xy}^{2\omega}$ on $(V_{xx}^{\omega})^2$ for the 1.40° AB-BA device.

Another characteristic feature of the nonlinear Hall effect is that when both the direction of current and voltage probes are reversed, $V_{xy}^{2\omega}$ picks up a negative sign. This behavior is depicted in Fig. S11a for the AB-AB device with I_ω on the x-axis, and in Fig. S12b with n on the x-axis for the AB-BA device.

In addition to the nonlinear Hall voltage ($V_{xy}^{2\omega}$), we also observed a nonlinear longitudinal voltage ($V_{xx}^{2\omega}$) in our TDBG (Fig. S11b for AB-AB and Fig. S12c for AB-BA TDBG) having twist angle of ≈1.4°. The source of the nonlinear longitudinal voltage is primarily attributed to extrinsic scattering mechanisms [11, 18, 19]. These include skew-scattering and side-jump scattering processes, which are influenced by disorder potentials in the system. A recent proposal also suggests an intrinsic mechanism that generates a nonzero dissipative longitudinal second-order voltage, governed by the Berry connection polarizability induced by quantum metric [20]. Here we note that the scaling analysis (discussed in the next section) that we use to extract the BCD by parametrically varying D/ϵ_0, is applicable to our devices with non-zero skew-scattering.

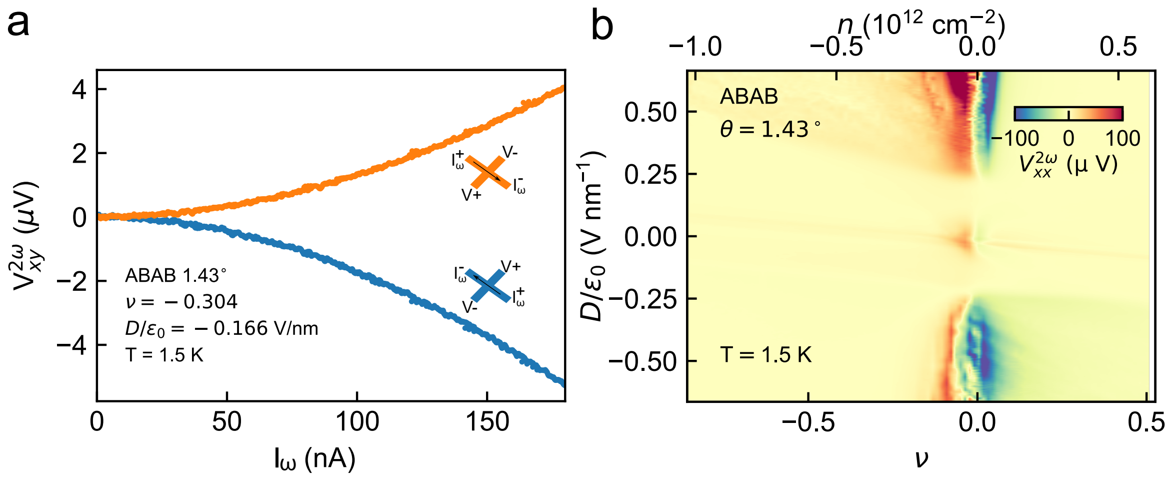

Fig. S11: Further characterisation of nonlinear voltage in AB-AB stacked TDBG device-1. a, Quadratic scaling of $V_{xy}^{2\omega}$ with channel current I_ω at a fixed ν and D/ϵ_0. The $V_{xy}^{2\omega}$ flips sign when the orientation of both the current and the voltage probes are flipped together. This indicates the second-order nature of the measured $V_{xy}^{2\omega}$. **b,** Colorscale plot of longitudinal nonlinear voltage $V_{xx}^{2\omega}$ as a function of ν and D/ϵ_0 close to the charge neutrality gap. The top axis shows the corresponding charge density n.

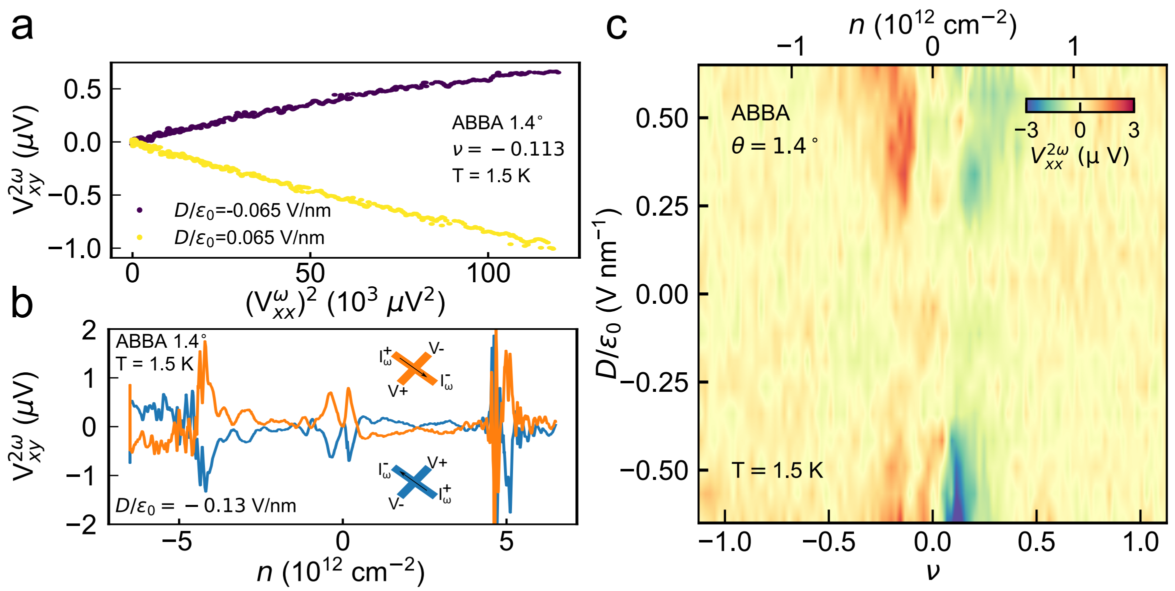

Fig. S12: Further characterization of nonlinear voltage in AB-BA stacked TDBG device. a, Variation of $V_{xy}^{2\omega}$ with $(V_{xx}^{\omega})^2$ for a fixed filling factor $\nu = -0.113$, for two different polarities of the perpendicular electric field D/ϵ_0. A linear behavior of $V_{xy}^{2\omega}$ with $(V_{xx}^{\omega})^2$ verifies the quadratic dependence of $V_{xy}^{2\omega}$ on current. **b,** $V_{xy}^{2\omega}$ vs n lineslice for a fixed D/ϵ_0, for two different configuration of current and voltage probes. The $V_{xy}^{2\omega}$ flips sign when the orientation of both the current and the voltage measuring probes are flipped together. This indicates the second-order nature of the measured $V_{xy}^{2\omega}$. **c,** Colorscale plot of longitudinal nonlinear voltage $V_{xx}^{2\omega}$ as a function of ν and D/ϵ_0 close to the charge neutrality gap. The top axis shows the corresponding charge density n.

VII Scaling Analysis and local intercept analysis

VII.1 Scaling Analysis

The nonlinear Hall voltage $V_{xy}^{2\omega}$ can originate from both the intrinsic (Berry curvature dipole) and the extrinsic mechanisms (such as side-jump scattering, skew-scattering). This is similar to the linear anomalous Hall voltage that can originate from both the intrinsic (Berry curvature) and the extrinsic scattering mechanisms [21]. Recent developments in the field suggest using a scaling relation to extract the Berry curvature dipole governed intrinsic contribution to $V_{xy}^{2\omega}$.

Accounting for all the mechanisms, a general scaling relation for the experimentally measured normalized NLH signal $\frac{V_{xy}^{2\omega}}{(V_{xx}^{\omega})^2}$, can be written as, [22]

$$\frac{V_{xy}^{2\omega}}{(V_{xx}^{\omega})^2} = \mathcal{C}^{in} + \sum_i \mathcal{C}_i^{sj}\frac{\rho_i}{\rho_{xx}} + \sum_{i,j}\mathcal{C}_{ij}^{sk1}\frac{\rho_i\rho_j}{\rho_{xx}^2} + \sum_i \mathcal{C}_i^{sk2}\frac{\rho_i}{\rho_{xx}^2}\ . \tag{12}$$

Here, i, j represent different sources of scattering. The coefficients stand for Berry curvature dipole ($\mathcal{C}^{in}$), side-jump ($\mathcal{C}^{sj}$), and skew-scattering ($\mathcal{C}^{sk}$) contributions (*sk1* and *sk2* represent two different

kinds of skew scattering). Considering only two sources of scattering, the static (impurities) and dynamic (phonon), we can express the above equation as

$$\frac{V_{xy}^{2\omega}}{(V_{xx}^{\omega})^2} = \frac{1}{\rho_{xx}^2}\left(\mathcal{C}_1\rho_{xx0} + \mathcal{C}_2\rho_{xx0}^2 + \mathcal{C}_3\rho_{xx0}\rho_{xxT} + \mathcal{C}_4\rho_{xxT}^2\right) \ . \tag{13}$$

Here, ρ_{xx0} is the zero temperature residual resistivity due to static impurities, and $\rho_{xxT} = \rho_{xx} - \rho_{xx0}$ is the contribution from phonons at finite temperature. The new parameter set in Eq. (13) can be obtained from the old one as

$$\mathcal{C}_1 = \mathcal{C}_0^{sk2}; \quad \mathcal{C}_2 = \mathcal{C}^{in} + \mathcal{C}_0^{sj} + \mathcal{C}_{00}^{sk1}, \tag{14}$$

$$\mathcal{C}_3 = 2\mathcal{C}^{in} + \mathcal{C}_0^{sj} + \mathcal{C}_1^{sj} + \mathcal{C}_{01}^{sk1}, \tag{15}$$

$$\mathcal{C}_4 = \mathcal{C}^{in} + \mathcal{C}_1^{sj} + \mathcal{C}_{11}^{sk1}. \tag{16}$$

Here, the indices 0 and 1 stand for static and phonon scattering sources, respectively. The coefficients $\mathcal{C}_{00}^{sk1}$, $\mathcal{C}_{11}^{sk1}$, $\mathcal{C}_{01}^{sk1}$ represent the skew-scattering contributions of the cross terms $\rho_i\rho_j/\rho_{xx}^2$ (where $i, j = 0, 1$) to the normalized NLH voltage in Eq. 12. ρ_i represents the resistivity contribution of i-th type of scattering source to the longitudinal resistivity $\rho_{xx} = \Sigma_i\rho_i$. We can rewrite the scaling relation in terms of the conductivity σ_{xx} as

$$\frac{V_{xy}^{2\omega}}{(V_{xx}^{\omega})^2} - \mathcal{C}_1\sigma_{xx0}^{-1}\sigma_{xx}^2 = (\mathcal{C}_2 + \mathcal{C}_4 - \mathcal{C}_3)\,\sigma_{xx0}^{-2}\sigma_{xx}^2 + (\mathcal{C}_3 - 2\mathcal{C}_4)\,\sigma_{xx0}^{-1}\sigma_{xx} + \mathcal{C}_4 \ . \tag{17}$$

Here, σ_{xx0} is the residual conductivity. After a rearrangement of the terms, Eq. 17 can be expressed as

$$\frac{V_{xy}^{2\omega}}{(V_{xx}^{\omega})^2} - \mathcal{C}_1\sigma_{xx0}^{-1}\sigma_{xx}^2 = (\mathcal{C}_2 - \mathcal{C}_4)\,\sigma_{xx0}^{-2}\sigma_{xx}^2 + (\mathcal{C}_3 - 2\mathcal{C}_4)\left(\sigma_{xx0}^{-1}\sigma_{xx} - \sigma_{xx0}^{-2}\sigma_{xx}^2\right) + \mathcal{C}_4 \ . \tag{18}$$

In the low-temperature limit in which we have done our experiment, the phonon contribution to the conductivity can be assumed to be small, and hence we consider $\sigma_{xx} \approx \sigma_{xx0}$. Consequently, the second term on the right-hand side $\left(\sigma_{xx0}^{-1}\sigma_{xx} - \sigma_{xx0}^{-2}\sigma_{xx}^2\right) \approx 0$ [22, 23]. This approximation allows

us to simplify Eq. 18 scaling relation as,

$$\frac{V_{xy}^{2\omega}}{(V_{xx}^{\omega})^2} = \left(\mathcal{C}_1\sigma_{xx0}^{-1} + \mathcal{C}_2\sigma_{xx0}^{-2} - \mathcal{C}_4\sigma_{xx0}^{-2}\right)\sigma_{xx}^2 + \mathcal{C}_4. \tag{19}$$

We use this simplified scaling relation for experimental fitting represented as

$$\frac{V_{xy}^{2\omega}}{(V_{xx}^{\omega})^2} = A\sigma_{xx}^2 + B\ , \tag{20}$$

where $A = \left(\mathcal{C}_1\sigma_{xx0}^{-1} + \mathcal{C}_2\sigma_{xx0}^{-2} - \mathcal{C}_4\sigma_{xx0}^{-2}\right) = \mathcal{C}_0^{sk2}\sigma_{xx0}^{-1} + \left(\mathcal{C}_0^{sj} + \mathcal{C}_{00}^{sk1} - \mathcal{C}_1^{sj} - \mathcal{C}_{11}^{sk1}\right)\sigma_{xx0}^{-2}$ represents the slope and $B = \mathcal{C}_4$ is the intercept. The intercept contains the BCD contribution, whereas the slope is solely determined by the skew-scattering and side-jump mechanism.

VII.2 D/ϵ_0 as a parameter to probe the scaling relation

One can perform the scaling analysis in Eq. 20 by varying experimentally controllable parameters such as temperature, perpendicular electric field, etc. In general, the scattering mechanisms, and hence, the scaling parameters A and B can be a function of temperature [22, 24]. We used the perpendicular electric field D/ϵ_0 as the parameter, at a fixed temperature, and performed the fitting within a suitable range of D/ϵ_0 where the BCD and scattering contributions remain relatively constant. This scaling relation allowed us to capture the BCD dependence on the polarity of D/ϵ_0 from the intercept η of $\frac{E_{xy}^{2\omega}}{(E_{xx}^{\omega})^2}=(L^2/w)\frac{V_{xy}^{2\omega}}{(V_{xx}^{\omega})^2}=\zeta\sigma_{xx}^2 + \eta$. Recent experiments report the observation of this scaling relation in different systems and use this analysis to estimate the order of magnitude of BCD, such as in few-layer WTe_2 [25], where BCD$\sim \eta E_F/e$. Here, E_F is the Fermi energy and is fixed experimentally by the filling factor ν to probe the scaling relation. To obtain the BCD dependence on the polarity of D/ϵ_0, the scaling is performed for a similar $|D/\epsilon_0|$ range for both $\pm$ D/ϵ_0 polarities, and the intercept is compared.

In TDBG, when the perpendicular electric field is varied over a large D/ϵ_0 range, the bands undergo valley Chern transitions (that is, a change in valley Chern numbers) that change the Berry curvature dipole. Therefore, we further performed the local intercept analysis as discussed below, and analyze the variation of the intercept over a large D/ϵ_0 range.

VII.3 Local Intercept analysis

We considered a small interval $(D_1/\epsilon_0,\ D_2/\epsilon_0)$ within which the BCD and scattering contributions can be considered unchanged. A straight line was drawn connecting $(\frac{E_{xy}^{2\omega}}{(E_{xx}^{\omega})^2}, \sigma_{xx}^2)|_{(D_1/\epsilon_0)}$ and $(\frac{E_{xy}^{2\omega}}{(E_{xx}^{\omega})^2}, \sigma_{xx}^2)|_{(D_2/\epsilon_0)}$ to extract the local intercept $\eta(D_1/\epsilon_0)$. This approach can be visualized as finding the tangent at D_1/ϵ_0. The local intercept of the tangent was then analyzed as a function of D/ϵ_0, with an interval, $D_1/\epsilon_0 -\ D_2/\epsilon_0 = 0.017\ \mathrm{V\,nm^{-1}}$ ($0.013\ \mathrm{V\,nm^{-1}}$) as shown in Fig. S14 (Fig. S18) at additional filling factor ν aside the one presented in the main manuscript, near the CNP for 1.43° AB-AB (1.4° AB-BA) TDBG. This method provides additional understanding of the nonlinear Hall voltage in TDBG by accounting for the dynamic changes in band structure when the D/ϵ_0 is varied substantially over a large range.

VIII Additional scaling data from AB-AB TDBG devices

VIII.1 Scaling data from AB-AB TDBG device-1 with twist angle 1.43°

In this subsection, we probe the scaling relation in 1.43° AB-AB TDBG for a few more filling factors, other than that shown in the main manuscript. We show the $\frac{E_{xy}^{2\omega}}{(E_{xx}^{\omega})^2}$ and σ_{xx}^2 as a function of D/ϵ_0 for $\nu = -0.055$ (Fig. S13a) and $\nu = -0.028$ (Fig. S13d). The peak in σ_{xx}^2 indicates band touching as discussed in the main manuscript. We probe the scaling of $\frac{E_{xy}^{2\omega}}{(E_{xx}^{\omega})^2}$ vs σ_{xx}^2 parametrically for both polarities of D/ϵ_0 (Fig. S13b,c for $\nu = -0.055$ and Fig. S13e,f for $\nu = -0.028$), in the D/ϵ_0 range just before the bands touch, similar to our analysis in Fig. 3d ($-$ve D/ϵ_0) and Fig. 3e (+ve D/ϵ_0) of the main manuscript. We note that as the polarity of D/ϵ_0 is flipped, the intercept reverses sign for both the fillings. To understand the behavior of the parametric plots for the full D/ϵ_0 range probed, we plot the local intercept over the extended D/ϵ_0 range in Fig. S14a ($\nu = -0.028$) and Fig. S14b ($\nu = -0.055$). We note the characteristic reversal in the intercept η, as the polarity of D/ϵ_0 is flipped, similar to that observed in Fig. 3f of the main manuscript. These observations further suggest that the BCD in 1.43° AB-AB TDBG flips sign with the polarity of D/ϵ_0.

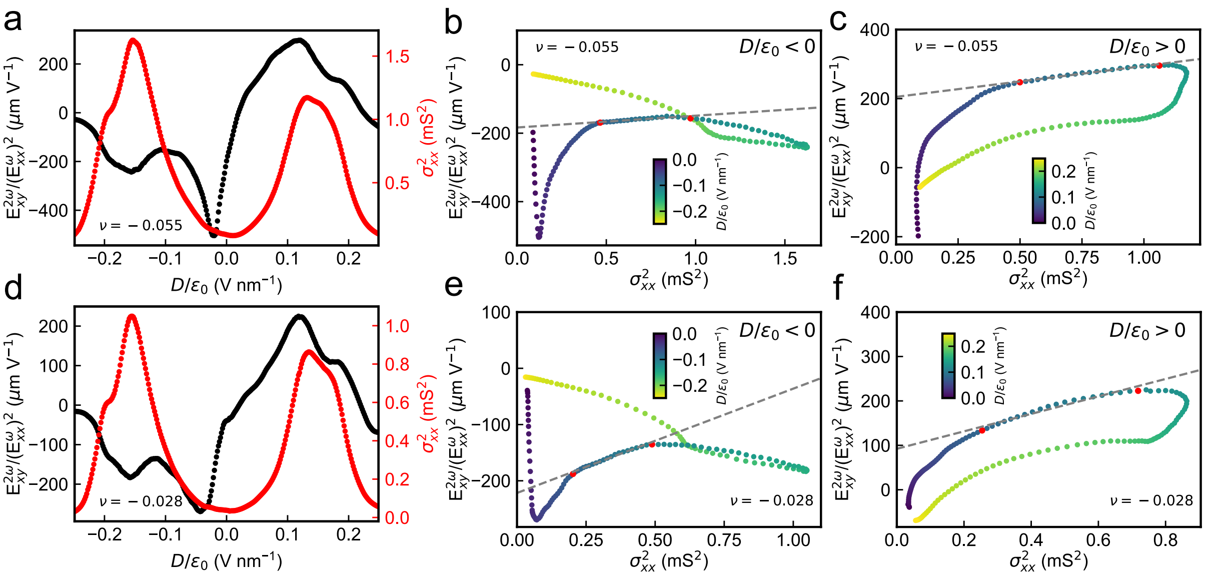

Fig. S13: Scaling of normalized nonlinear Hall signal $E_{xy}^{2\omega}/(E_{xx}^{\omega})^2$ with the square of longitudinal conductivity (σ_{xx}^2) for different fillings ν in 1.43° AB-AB TDBG device. a-c, For filling $\nu = -0.055$ and **d-e,** for $\nu = -0.028$. **a,d,** Variation of normalized nonlinear Hall signal $E_{xy}^{2\omega}/(E_{xx}^{\omega})^2$ (black colored data points corresponding to the left axis) and square of longitudinal conductivity σ_{xx}^2 (red-colored data points corresponding to the right axis) as a function of the displacement field D/ϵ_0. **b,c,** and **e,f,** Parametric plots of the normalized nonlinear Hall signal $E_{xy}^{2\omega}/(E_{xx}^{\omega})^2$ against the square of the longitudinal conductivity σ_{xx}^2 corresponding to data from **a** and **d** respectively, for $D/\epsilon_0<0$ (**b,e**) and $D/\epsilon_0>0$ (**c,f**). The color bar indicates the displacement field value of data points in V nm^{-1}. We note that the sign of the intercept changes for each of the fillings, as the polarity of D/ϵ_0 is flipped. The measurements were performed using a current of 100 nA with a frequency of 177 Hz at a temperature of 1.2 K.

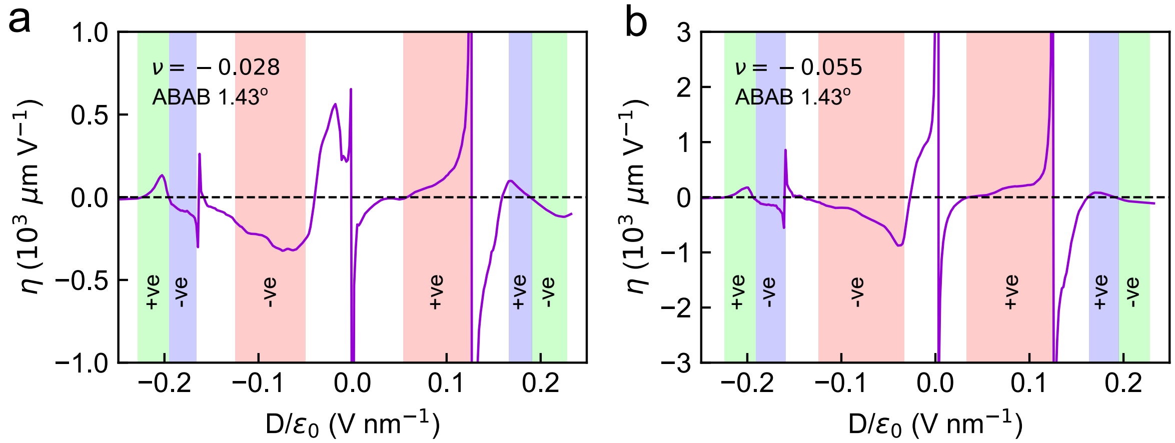

Fig. S14: Variation of local intercept over a large range of perpendicular electric field in AB-AB TDBG device-1 at different filling factor ν. a, b, The local intercept η, defined for a small moving D/ϵ_0 range of 0.017 V nm^{-1}, extracted as a function of D/ϵ_0 from the data presented in figure S13d and S13a for $\nu = -0.028$ (a) and $\nu = -0.055$ (b) respectively. The colors indicate the different D/ϵ_0 ranges where η, and hence BCD, flip sign across $D = 0$. The temperature was $T = 1.2$ K.

VIII.2 Scaling data from AB-AB TDBG device-2 with twist angle 1.1°

In Fig. S15a and Fig. S15b, we show the colorscale plot of R_{xx} and the $V_{xy}^{2\omega}$, respectively, as a function of ν and D/ϵ_0. In Fig. S15c and Fig. S15d, we show the corresponding $\frac{E_{xy}^{2\omega}}{(E_{xx}^{\omega})^2}$, σ_{xx}^2 vs. D/ϵ_0 dependence with a fixed filling factor near the charge neutrality when the gap between the flat bands start opening [1], for positive and negative polarity of D/ϵ_0 respectively. Fig. S15e and Fig. S15f probe the corresponding scaling of $\frac{E_{xy}^{2\omega}}{(E_{xx}^{\omega})^2}$ with σ_{xx}^2 for positive and negative D/ϵ_0. We note that the intercept flips sign as the polarity of D/ϵ_0 is flipped.

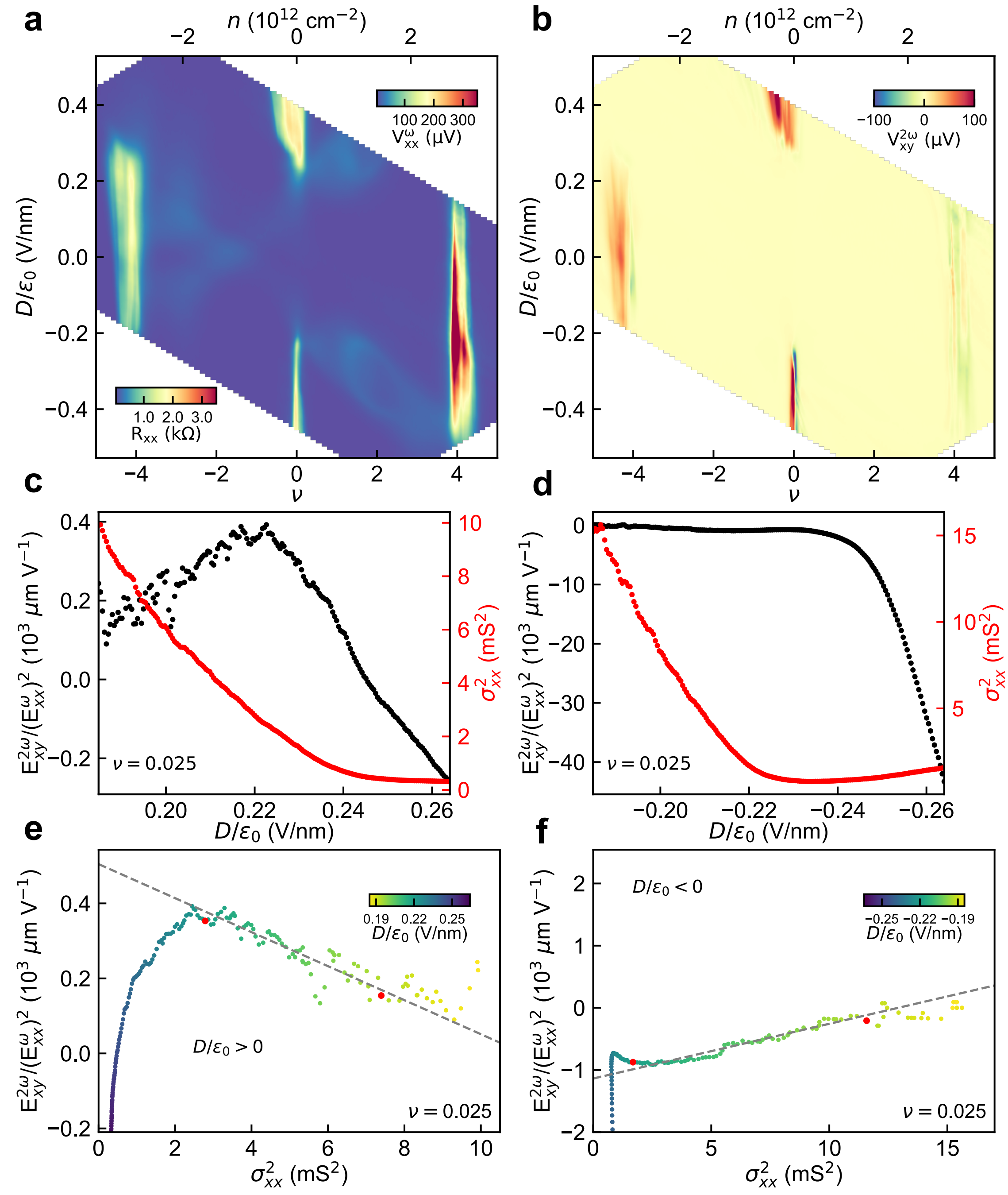

Fig. S15: Scaling of normalized nonlinear Hall voltage $V_{xy}^{2\omega}/(V_{xx}^{\omega})^2$ with the square of longitudinal conductivity (σ_{xx}^2) in 1.1° AB-AB TDBG device-2. a,b, R_{xx} (**a**) and NLH voltage $V_{xy}^{2\omega}$ (**b**) as a function of ν and D/ϵ_0 of 1.1° AB-AB TDBG device. The top x-axis shows the corresponding n values. **c,d,** The variation of normalized nonlinear Hall signal $E_{xy}^{2\omega}/(E_{xx}^{\omega})^2$ (black colored data points corresponding to the left axis) and square of longitudinal conductivity σ_{xx}^2 (red-colored data points corresponding to the right axis) as a function of the displacement field D/ϵ_0, for a fixed filling close to the charge neutrality gap. **e,f,** The variation of normalized nonlinear Hall signal $E_{xy}^{2\omega}/(E_{xx}^{\omega})^2$ with square of longitudinal conductivity σ_{xx}^2 plotted parametrically as a function of the displacement field D/ϵ_0, using **c**, and **d**, respectively, for $D/\epsilon_0>0$ (**e**) and $D/\epsilon_0<0$ (**f**). The color bar indicates the displacement field value of data points in V nm^{-1}. We note that the sign of the intercept flips, as the polarity of D/ϵ_0 is flipped. The measurements were performed using a current of 100 nA with a frequency of 177 Hz at a temperature of 1.5 K.

IX Additional scaling data from AB-BA TDBG device

In this section, we probe the D/ϵ_0 polarity dependence of the scaling relation for a few more filling factors other than that shown in the main manuscript, for the 1.4° AB-BA TDBG device. Fig. S16a, b, and c shows the measured nonlinear Hall voltage $V_{xy}^{2\omega}$ and longitudinal voltage V_{xx}^{ω} as a function of D/ϵ_0 for three different $\nu = -0.214, -0.259, -0.270$. Using the measured V_{xx}^{ω} and $V_{xy}^{2\omega}$, we plot the corresponding normalized NLH electric field $\frac{E_{xy}^{2\omega}}{(E_{xx}^{\omega})^2}$ and $\sigma_{xx}^2 = (\mathrm{I}/V_{xx}^{\omega})^2$ as a function of D/ϵ_0 for the three fillings in Fig. S16d, e, and f. In Fig. S16g-l, we plot the $\frac{E_{xy}^{2\omega}}{(E_{xx}^{\omega})^2}$ vs. σ_{xx}^2 parametrically with D/ϵ_0, for D/ϵ_0<0 (Fig. S16g-i) and D/ϵ_0>0 (Fig. S16j-l). We probe the scaling for the low D/ϵ_0 range just before the conductivity maximizes, for both polarities of D/ϵ_0. We observe a linear scaling in a similar range of $|D/\epsilon_0|$ range for both the polarities of D/ϵ_0. We observe that the sign of the intercept (η) remains the same as the polarity of D/ϵ_0 is flipped, and the extracted magnitude of η decreases as one moves away from the band edge into the valence band (Fig. S17). This BCD dependence is consistent with that extracted in Fig. 4d of Zhong, J. et al. [26]. In Fig. S18b ($\nu = 0$) and Fig. S18d ($\nu = -0.068$), we further show the local intercept over the extended D/ϵ_0 range for two fillings close to the charge neutrality gap. We note that the intercept η does not flip the sign in similar D/ϵ_0 ranges indicated by the same color, as the polarity of D/ϵ_0 is flipped, like that observed in Fig. 4f of the main manuscript. These observations further suggest that the BCD in 1.4° AB-BA TDBG device does not flip sign with the polarity of D/ϵ_0.

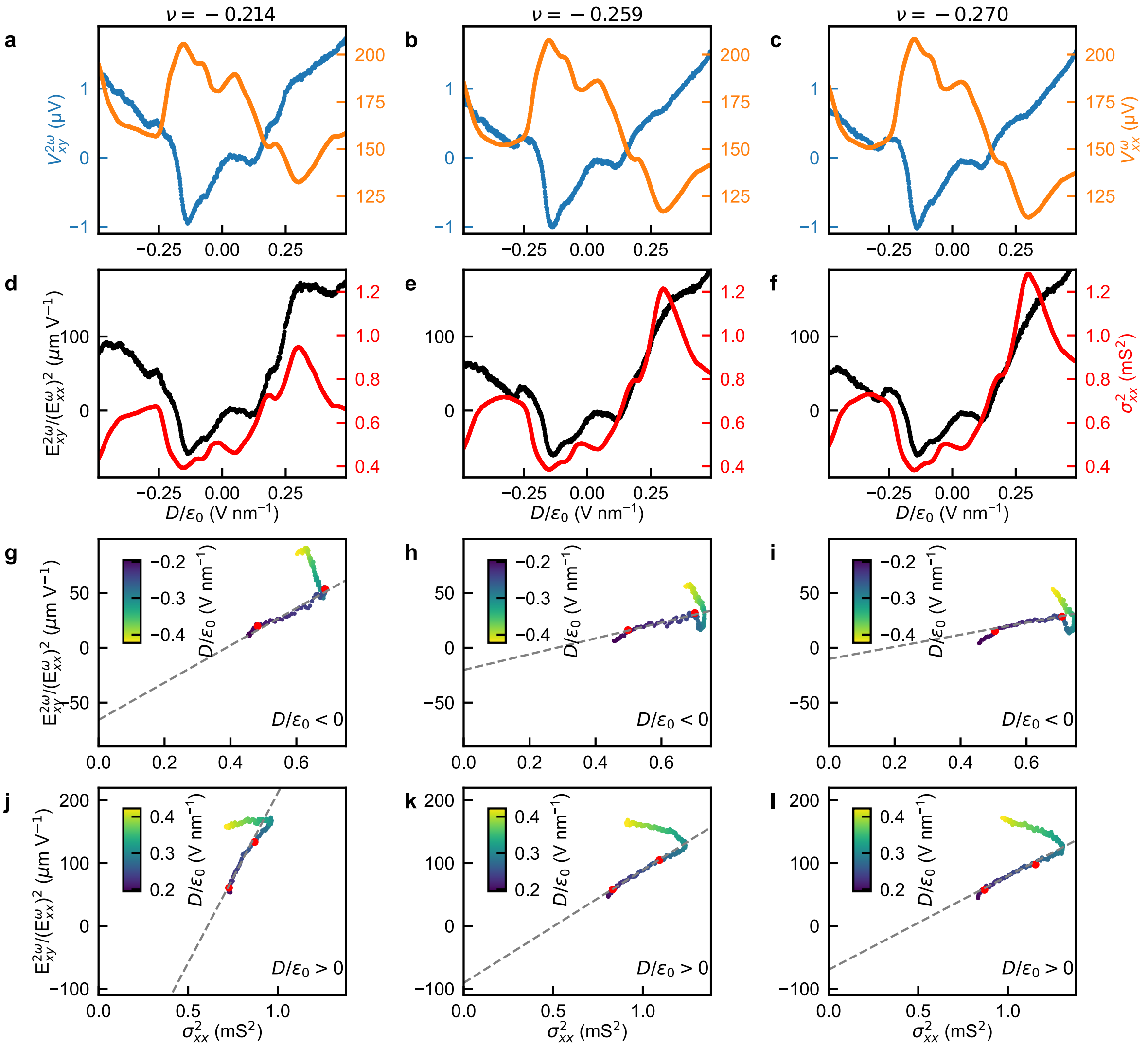

Fig. S16: Scaling of normalized nonlinear Hall voltage $V_{xy}^{2\omega}/(V_{xx}^{\omega})^2$ with the square of longitudinal conductivity (σ_{xx}^2) for different fillings ν in 1.4° AB-BA TDBG device. a-c, The variation of nonlinear Hall voltage $V_{xy}^{2\omega}$ (blue-colored data points corresponding to the left axis) and longitudinal voltage V_{xx}^{ω} (orange-colored data points corresponding to the right axis) as a function of the displacement field D/ϵ_0 for three different fillings ν (different from those presented in Fig. 4 of the main manuscript). **d-f,** The corresponding variation of normalized nonlinear Hall signal $E_{xy}^{2\omega}/(E_{xx}^{\omega})^2$ (black colored data points corresponding to the left axis) and square of longitudinal conductivity σ_{xx}^2 (red-colored data points corresponding to the right axis) as a function of the displacement field D/ϵ_0, extracted for the same fillings used in **a**, **b**, and **c**, respectively. **g-l,** The variation of normalized nonlinear Hall signal $E_{xy}^{2\omega}/(E_{xx}^{\omega})^2$ with square of longitudinal conductivity σ_{xx}^2 plotted parametrically as a function of the displacement field D/ϵ_0, using **d**, **e**, and **f**, respectively, for $D/\epsilon_0<0$ (**g-i**) and $D/\epsilon_0>0$ (**j-l**). The color bar indicates the displacement field value of data points in V nm^{-1}. We note that the sign of the intercept remains the same for each of the fillings, as the polarity of D/ϵ_0 is flipped. The measurements were performed using a current of 100 nA with a frequency of 177 Hz at a temperature of 1.5 K.

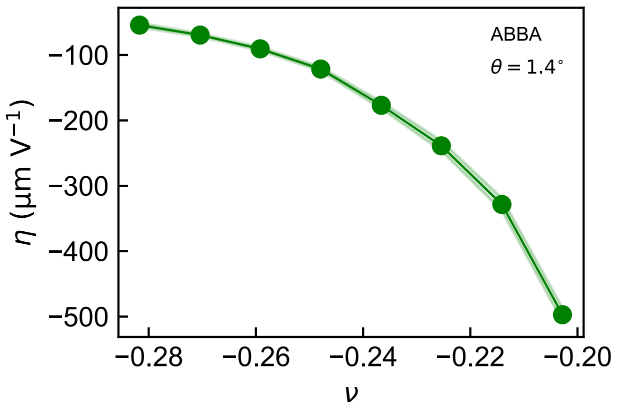

Fig. S17: Extracted intercept as a function of filling factor (ν) for the valence band and positive perpendicular electric field (D/ϵ_0) in AB-BA TDBG with a twist angle of 1.4°. The BCD (Λ) $\sim \eta E_F/e$, decreases in magnitude as the filling factor moves away from the band edge. Individual fittings are shown in Fig. S16

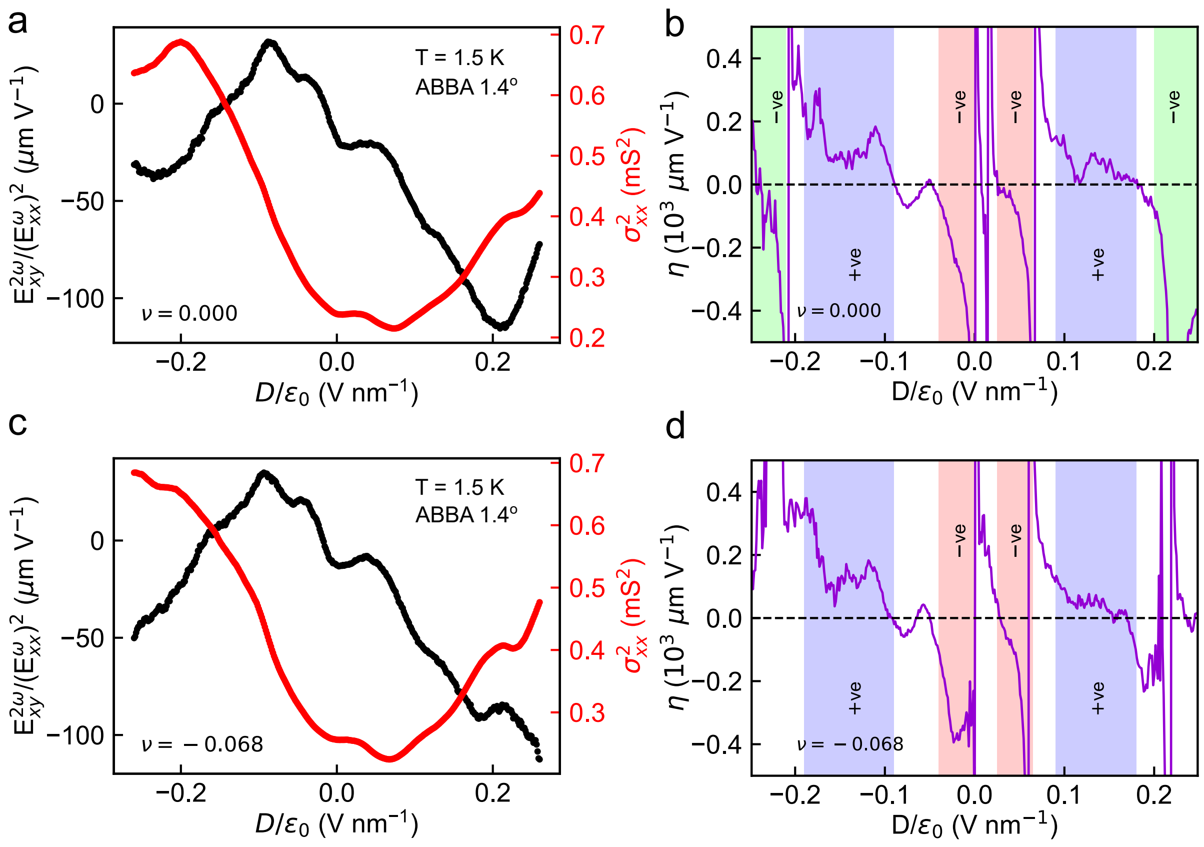

Fig. S18: Variation of local intercept over a large range of perpendicular electric field in AB-BA TDBG device at different filling factor ν. a,c, Variation of normalized nonlinear Hall signal $E^{2\omega}_{xy}/(E^{\omega}_{xx})^2$ (black colored data points corresponding to the left axis) and square of longitudinal conductivity σ^2_{xx} (red-colored data points corresponding to the right axis) as a function of the displacement field D/ϵ_0 for $\nu = 0.0$ (a) and $\nu = -0.068$ (c). **b, d,** The local intercept η, defined for a small moving D/ϵ_0 range of 0.013 V nm^{-1}, extracted as a function of D/ϵ_0 from the data in **a** and **c** respectively. The colors indicate the different D/ϵ_0 ranges where η, and hence BCD, has same sign across $D = 0$. The temperature was $T = 1.5$ K.

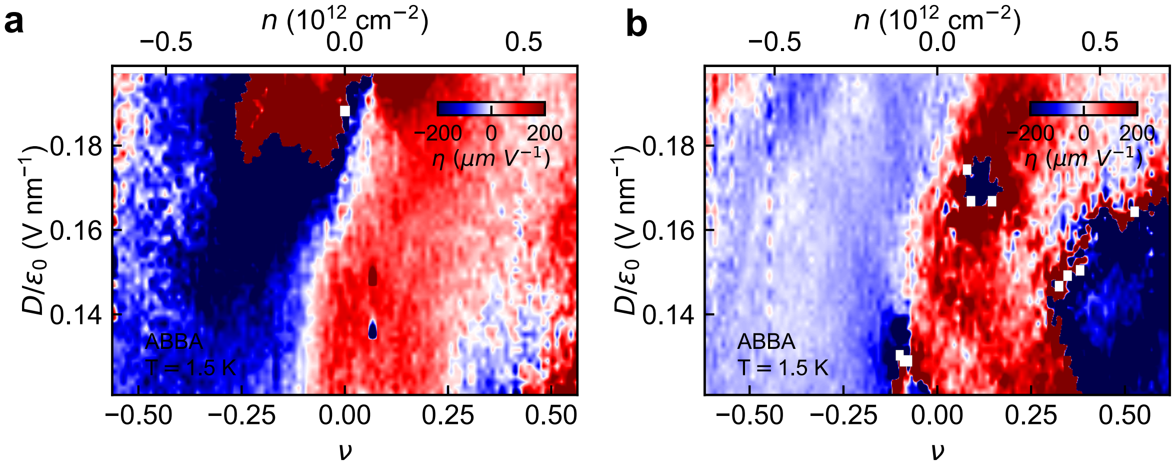

Fig. S19: Extracted intercept η as a function of filling factor (ν) and the perpendicular electric field (D/ϵ_0) for AB-BA TDBG with a twist angle of $\sim 1.4^\circ$ for two different pairs of probe combinations. The BCD (Λ) $\sim \eta E_F/e$, flips sign across $\nu = 0$, that is, as one moves from the valence band to the conduction band. This sign flip is consistent with the theoretically calculated BCD sign flip in Fig. 4a of the main manuscript across E=8 meV.

References

[1] Sinha, S. *et al.* Berry curvature dipole senses topological transition in a moiré superlattice. *Nature Physics* **18**, 765–770 (2022).

[2] Chakraborty, A., Dutta, D. & Agarwal, A. Tunable interband and intraband plasmons in twisted double bilayer graphene. *Phys. Rev. B* **106**, 155422 (2022).

[3] Chakraborty, A. *et al.* Nonlinear anomalous hall effects probe topological phase-transitions in twisted double bilayer graphene. *2D Materials* **9**, 045020 (2022).

[4] Dutta, D., Chakraborty, A. & Agarwal, A. Intrinsic nonreciprocal bulk plasmons in noncentrosymmetric magnetic systems. *Phys. Rev. B* **107**, 165404 (2023).

[5] Koshino, M. Band structure and topological properties of twisted double bilayer graphene. *Physical Review B* **99**, 235406 (2019).

[6] Chebrolu, N. R., Chittari, B. L. & Jung, J. Flat bands in twisted double bilayer graphene. *Phys. Rev. B* **99**, 235417 (2019).

[7] Mohan, P., Ghorai, U. & Sensarma, R. Trigonal warping, satellite dirac points, and multiple field tuned topological transitions in twisted double bilayer graphene. *Phys. Rev. B* **103**, 155149 (2021).

[8] He, W.-Y., Goldhaber-Gordon, D. & Law, K. T. Giant orbital magnetoelectric effect and current-induced magnetization switching in twisted bilayer graphene. *Nature Communications* **11**, 1650 (2020).

[9] Low, T., Guinea, F. & Pantaleón, P. A. Tunable large Berry dipole in strained twisted bilayer graphene. *Physical Review B* **103**, 205403 (2021).

[10] Schaefer, B. T. & Nowack, K. C. Electrically tunable and reversible magnetoelectric coupling in strained bilayer graphene. *Phys. Rev. B* **103**, 224426 (2021).

[11] Datta, S. *et al.* Nonlinear electrical transport unveils fermi surface malleability in a moiré heterostructure. *Nano Letters* **24**, 9520–9527 (2024).

[12] Junaid, M. & Witjaksono, G. Analysis of band gap in aa and ab stacked bilayer graphene by hamiltonian tight binding method. In *2019 IEEE International Conference on Sensors and Nanotechnology*, 1–4 (2019).

[13] Shimazaki, Y. *et al.* Generation and detection of pure valley current byăelectrically induced berry curvature in bilayerăgraphene. *Nature Physics* **11**, 1032–1036 (2015).

[14] Varma Sangani, L. D. *et al.* Facile deterministic cutting of 2D materials for twistronics using a tapered fibre scalpel. *Nanotechnology* **31**, 32LT02 (2020).

[15] Adak, P. C. *et al.* Perpendicular electric field drives Chern transitions and layer polarization changes in Hofstadter bands. *Nature Communications* **13**, 7781 (2022).

[16] Kazmierczak, N. P. *et al.* Strain fields in twisted bilayer graphene. *Nature Materials* **20**, 956–963 (2021).

[17] McGilly, L. J. *et al.* Visualization of moiré superlattices. *Nature Nanotechnology* **15**, 580–584 (2020).

[18] Duan, J. *et al.* Giant Second-Order Nonlinear Hall Effect in Twisted Bilayer Graphene. *Physical Review Letters* **129**, 186801 (2022).

[19] He, P. *et al.* Graphene moiré superlattices with giant quantum nonlinearity of chiral Bloch electrons. *Nature Nanotechnology* **17**, 378–383 (2022).

[20] Das, K., Lahiri, S., Atencia, R. B., Culcer, D. & Agarwal, A. Intrinsic nonlinear conductivities induced by the quantum metric. *Phys. Rev. B* **108**, L201405 (2023).

[21] Nagaosa, N., Sinova, J., Onoda, S., MacDonald, A. H. & Ong, N. P. Anomalous Hall effect. *Reviews of Modern Physics* **82**, 1539–1592 (2010).

[22] Du, Z. Z., Wang, C. M., Li, S., Lu, H.-Z. & Xie, X. C. Disorder-induced nonlinear Hall effect with time-reversal symmetry. *Nature Communications* **10**, 3047 (2019).

[23] Huang, M. *et al.* Giant nonlinear Hall effect in twisted bilayer WSe_2. *National Science Review* nwac232 (2022).

[24] Xiao, C., Zhou, H. & Niu, Q. Scaling parameters in anomalous and nonlinear Hall effects depend on temperature. *Physical Review B* **100**, 161403 (2019).

[25] Kang, K., Li, T., Sohn, E., Shan, J. & Mak, K. F. Nonlinear anomalous Hall effect in few-layer WTe_2. *Nature Materials* **18**, 324–328 (2019).

[26] Zhong, J. *et al.* Effective Manipulation of a Colossal Second-Order Transverse Response in an Electric-Field-Tunable Graphene Moiré System. *Nano Letters* **24**, 5791–5798 (2024).
